# Association Between Computed Tomography–Based AI-Derived Body Composition and Survival in Patients With Pancreatic Ductal Adenocarcinoma

**DOI:** 10.14309/ajg.0000000000003896

**Published:** 2025-12-22

**Authors:** Koen J.H. Wijsman, Derk C.F. Klatte, Hani M. Babiker, Aleksander M. Bogdanski, Brandon R. Grossardt, Jeanin E. van Hooft, Monique E. van Leerdam, J. Sven D. Mieog, Alexander D. Weston, Michael B. Wallace, Yan Bi

**Affiliations:** 1 Department of Gastroenterology and Hepatology, Leiden University Medical Center, Leiden, the Netherlands;; 2 Division of Gastroenterology and Hepatology, Department of Medicine, Mayo Clinic, Jacksonville, Florida, USA;; 3 Division of Hematology and Oncology, Department of Medicine, Mayo Clinic, Jacksonville, Florida, USA;; 4 Department of Quantitative Health Sciences, Mayo Clinic, Rochester, Minnesota, USA;; 5 Department of Gastrointestinal Oncology, Netherlands Cancer Institute, Amsterdam, the Netherlands;; 6 Department of Surgery, Leiden University Medical Center, Leiden, the Netherlands;; 7 Department of Quantitative Health Sciences, Mayo Clinic, Jacksonville, Florida, USA.

**Keywords:** pancreatic ductal adenocarcinoma, body composition, survival analysis, mortality, artificial intelligence

## Abstract

**INTRODUCTION:**

A deeper understanding of the factors influencing survival in patients with pancreatic ductal adenocarcinoma (PDAC) is essential for optimizing treatment strategies. This study investigates the independent association of body composition parameters with overall survival in patients with PDAC.

**METHODS:**

This retrospective multisite cohort study included patients diagnosed with PDAC. Diagnostic computed tomography scans were retrieved, and body composition was evaluated using a validated deep learning-based segmentation algorithm that measured tissue volume and density in a 20 cm vertical abdominal section.

**RESULTS:**

A total of 1,666 patients with PDAC were included, 938 male (56.3%) and median age 69 years (interquartile range 61–76). In the subgroup of patients who underwent surgical resection (n = 509), myosteatosis (intramuscular infiltration of adipose tissue; hazard ratio [HR] 1.56, 95% confidence interval [CI] 1.16–2.11, *P* = 0.004), sarcopenic obesity (HR 1.75, 95% CI 1.06–2.91, *P* = 0.03), and less subcutaneous adipose tissue (HR 1.09, 95% CI 1.03–1.16, *P* = 0.002) were associated with higher mortality. In patients receiving palliative systemic therapy (n = 439), lower skeletal muscle density (HR 1.43, 95% CI 1.03–1.99, *P* = 0.03) was associated with higher mortality. In patients who did not undergo tumor-targeted treatment (n = 718), less visceral adipose tissue (HR 1.04, 95% CI 1.01–1.08, *P* = 0.02) was associated with higher mortality.

**DISCUSSION:**

Body composition parameters, derived from computed tomography scans at the time of PDAC diagnosis, particularly low skeletal muscle density, sarcopenic obesity, and low adipose tissue, are independently associated with overall survival in patients with PDAC. Evaluating body composition at diagnosis could enhance clinical decision-making and enable more personalized treatment strategies.

## Graphical abstract

**Figure:**
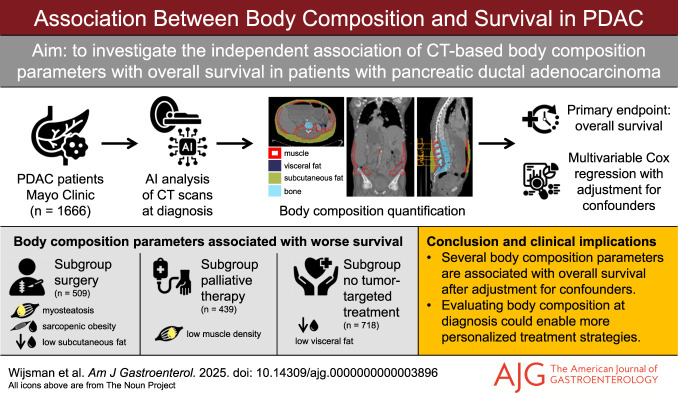

## INTRODUCTION

Pancreatic ductal adenocarcinoma (PDAC) is among the deadliest cancers, currently the third leading cause of cancer-related deaths in the United States (1), with projections to become the second by 2030 (2). Surgical resection offers the only potential cure, but comes with significant morbidity, a 1.7% mortality rate (3) and a 76.7% recurrence rate, with a median recurrence-free survival of 12 months (4). However, surgery is not an option for most patients because 30%–35% present with locally advanced unresectable disease and 50%–55% with metastatic disease (5). For these patients, palliative chemotherapy is the primary treatment, but their low performance status combined with the substantial burden of treatment leads to 68% of these patients not receiving tumor-targeted treatment (6).

Although surgery can be life-saving and palliative therapy can be life-prolonging, these treatments are often burdensome and do not always yield substantial survival benefits. This underscores the importance of carefully selecting patients who are most likely to benefit from these treatments. Conversely, among patients who do not undergo tumor-targeted treatment and receive best supportive care, identifying those who could still benefit from palliative therapy may offer survival benefits. A better understanding of the factors influencing prognosis in PDAC could aid in personalizing treatment strategies.

Prognosis in PDAC is influenced by both tumor-specific factors and patient-specific factors, such as comorbidities, performance status, and body composition (7). While body composition has traditionally been assessed using body mass index (BMI), this measure is insufficient because it does not differentiate between tissue types. An increasing body of literature recognizes a more comprehensive assessment of abdominal body composition (defined as the quantity and density of skeletal muscle, visceral adipose tissue, subcutaneous adipose tissue, and bone within the abdomen) as an important factor influencing overall survival (OS) in patients with PDAC (8). For instance, sarcopenia (decreased muscle mass) (9–14), sarcopenic obesity (combined presence of sarcopenia and obesity) (7,15–20), myosteatosis (reduced muscle quality because of intramuscular infiltration of ectopic adipose tissue) (19,21–31), high visceral adipose tissue (29,30,32,33), and high subcutaneous adipose tissue (33) have been associated with worse OS. However, studies often yield contradictory results, potentially because of ethnic variability, differences in assessment techniques and cut-off values, and small sample sizes, which limit the generalizability of their findings.

Currently, abdominal body composition is often estimated by segmenting a single computed tomography (CT) slice at the level of the third lumbar vertebra (L3) into different tissue types. However, recent advancements in artificial intelligence now enable a more accurate assessment of body composition across a larger abdominal section (34). This study is among the first to utilize a deep learning-based algorithm to extract 3D abdominal body composition parameters from routinely performed diagnostic CT scans of patients with PDAC. Our objective was to investigate the independent association of body composition parameters with OS, aiming to better understand how these parameters influence the prognosis of patients with PDAC.

## METHODS

### Study design and population

This retrospective cohort study identified patients (18 years or older) diagnosed with PDAC from 2000 to 2020 at Mayo Clinic sites in Minnesota, Florida, and Arizona. Exclusion criteria were the absence of an abdominal CT scan at the day of or within 1 month before initial clinical PDAC diagnosis, lack of histopathological confirmation of PDAC, and the presence of other synchronous neoplasms. This study was conducted in accordance with the Strengthening the Reporting of Observational studies in Epidemiology (STROBE) guidelines (35). The study was approved by the Mayo Clinic Institutional Review Board (IRB protocol #21-011051).

### Data collection

Demographic (age, sex, race/ethnicity), lifestyle (alcohol consumption, smoking status), clinical (Eastern Cooperative Oncology Group [ECOG] performance status, comorbidities), tumor (localization, stage), laboratory (carbohydrate antigen 19-9 [CA19-9]), treatment, and survival data were collected from the Mayo Clinic Cancer Registry and patient medical records. Pre-existing comorbidities were aggregated into a modified version of the Charlson Comorbidity Index (CCI), excluding age, diabetes, and tumor stage because these factors were separately adjusted for in the regression analyses.

### Measurement of body composition parameters

**Figure 1.**
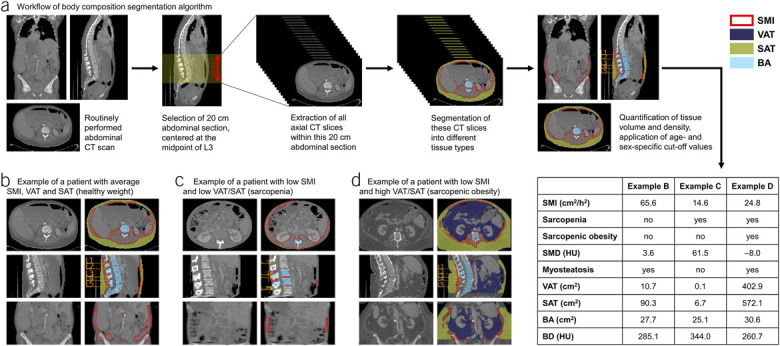
Overview of the body composition segmentation algorithm and representative examples of patients. (**a**) Body composition was derived from CT scans using a segmentation algorithm that automatically selects a 20 cm section of the abdomen, centered at the midpoint of the third lumbar vertebra (L3). Within this section, all axial CT slices are segmented into different tissue types, after which the quantity and density of these tissue types are measured. Skeletal muscle tissue is outlined in red, visceral adipose tissue is colored purple, subcutaneous adipose tissue is colored green, and bone tissue is colored blue. (**b**) Example of a patient with average SMI, VAT, and SAT (healthy weight). (**c**) Example of a patient with low SMI and low VAT and SAT (sarcopenia). (**d**) Example of a patient with low SMI and high VAT and SAT (sarcopenic obesity). BA, bone area; BD, bone density; CT, computed tomography; SAT, subcutaneous adipose tissue area; SMD, skeletal muscle density; SMI, skeletal muscle index; VAT, visceral adipose tissue area.

Body composition parameters were extracted from diagnostic CT scans using a previously validated deep learning-based segmentation algorithm, which uses a 3-dimensional U-Net Convolutional Neural Network model (Figure 1) that achieved a Dice similarity coefficient of 0.92–0.98 (36). Detailed information on the training, validation, and functionality of the algorithm is outlined in the Supplementary Digital Content (see Supplementary Methods, http://links.lww.com/AJG/D845) and a previous publication (36). The algorithm automatically selects a 20 cm vertical section of the abdomen, centered at the midpoint of the L3 vertebra, and reports the average skeletal muscle area (SMA, cm^2^), visceral adipose tissue area (VAT, cm^2^), subcutaneous adipose tissue area (SAT, cm^2^), and bone area (BA, cm^2^) within this section. To account for differences in stature, SMA was indexed to the patient's height in meters squared, resulting in the skeletal muscle index (SMI, cm^2^/m^2^). Skeletal muscle density (SMD) and bone density (BD) were defined as the average Hounsfield unit (HU) attenuation of all voxels within their respective compartments, with lower HU values indicating lower tissue radiodensity. All abdominal segmentations generated by the body composition algorithm were manually reviewed by an expert (A.D.W.) to ensure accurate segmentation.

### Definitions of obesity, sarcopenia, sarcopenic obesity, and myosteatosis

Height data (missing for 13 patients, 0.8%) and weight data (missing for 174 patients, 10.4%) were imputed using Multivariate Imputation by Chained Equations. BMI was calculated, and obesity was defined as BMI ≥30 kg/m^2^. Age- and sex-specific cut-off values for sarcopenia (based on SMI) and myosteatosis (based on SMD) were derived from a previous study that used the same abdominal segmentation algorithm to establish reference ranges for CT-based body composition parameters in a population-representative American cohort (see Supplementary Methods and Supplementary Table 1, http://links.lww.com/AJG/D845) (37). SMD below the age-specific and sex-specific threshold serves as a proxy for myosteatosis (decreased muscle quality due to intramuscular infiltration of ectopic adipose tissue), as adipose tissue has a lower radiodensity than muscle tissue (38). Notably, sarcopenia is not limited to individuals of normal or low weight; it also affects overweight individuals. This phenomenon, termed sarcopenic obesity, was defined as the combined presence of sarcopenia and obesity (39).

### Statistical analysis

The primary objective of this study was to investigate the association of various CT-based body composition parameters with OS, defined as the time from PDAC diagnosis to death from any cause. Patients who were still alive at the time of analysis were censored at the date of their last recorded contact. Hazard ratios (HRs) for mortality and 95% confidence intervals (CIs) were calculated using univariable and multivariable Cox proportional hazards regression models. To examine the independent association between body composition parameters and OS, separate multivariable analyses were conducted, adjusting each body composition parameter individually for potential confounders (age, sex, race/ethnicity, alcohol consumption, smoking status, ECOG Performance Status, BMI, comorbidities, diabetes, tumor localization, tumor stage, and CA19-9 level). Analyses were performed using both continuous muscle variables (SMI and SMD) and their corresponding dichotomous variables (sarcopenia and myosteatosis). While we recognize that continuous variables provide more detailed information, dichotomous classifications are more intuitive for clinical interpretation. Kaplan-Meier plots were used to estimate cumulative OS, and the log-rank test was performed to compare OS between groups. Statistical analyses were conducted using R (version 4.3.2). All *P* values were 2-sided, and *P* < 0.05 was considered statistically significant.

## RESULTS

### Patient characteristics and body composition parameters

A total of 1,825 patients diagnosed with PDAC were identified from the Mayo Clinic Cancer Registry. Of these, 127 patients were excluded due to the absence of a CT scan within 1 month before diagnosis, and 32 were excluded due to a lack of histopathological confirmation of PDAC. Of the included 1,666 patients, 938 (56.3%) were male, and the median age was 69 years (interquartile range 61–76). Demographic characteristics, clinical characteristics, and body composition parameters of the study population are summarized in Table 1 and Supplementary Digital Content (see Supplementary Table 2, http://links.lww.com/AJG/D845). Sarcopenia was present in 772 patients (46.3%), sarcopenic obesity in 69 patients (4.1%), and myosteatosis in 328 patients (19.7%). A detailed breakdown of the CCI is provided in Supplementary Digital Content (see Supplementary Table 3, http://links.lww.com/AJG/D845). Supplementary Digital Content (see Supplementary Table 4, http://links.lww.com/AJG/D845) summarizes treatment characteristics, including surgery details and margins, chemotherapy, radiotherapy, and treatment sequencing. Supplementary Digital Content (see Supplementary Table 5, http://links.lww.com/AJG/D845) consists of detailed imaging acquisition and reconstruction parameters. The study population was stratified into 3 subgroups based on treatment type: 509 patients (30.6%) who underwent curative-intent surgery, 439 patients (26.4%) who received palliative therapy (i.e., systemic therapy or radiation therapy), and 718 patients (43.1%) who did not undergo tumor-targeted treatment (i.e., best supportive care). Survival outcomes for these subgroups are presented in Supplementary Digital Content (see Supplementary Figure 1, http://links.lww.com/AJG/D845). Further survival analysis was performed separately within these subgroups.

**Table 1.**
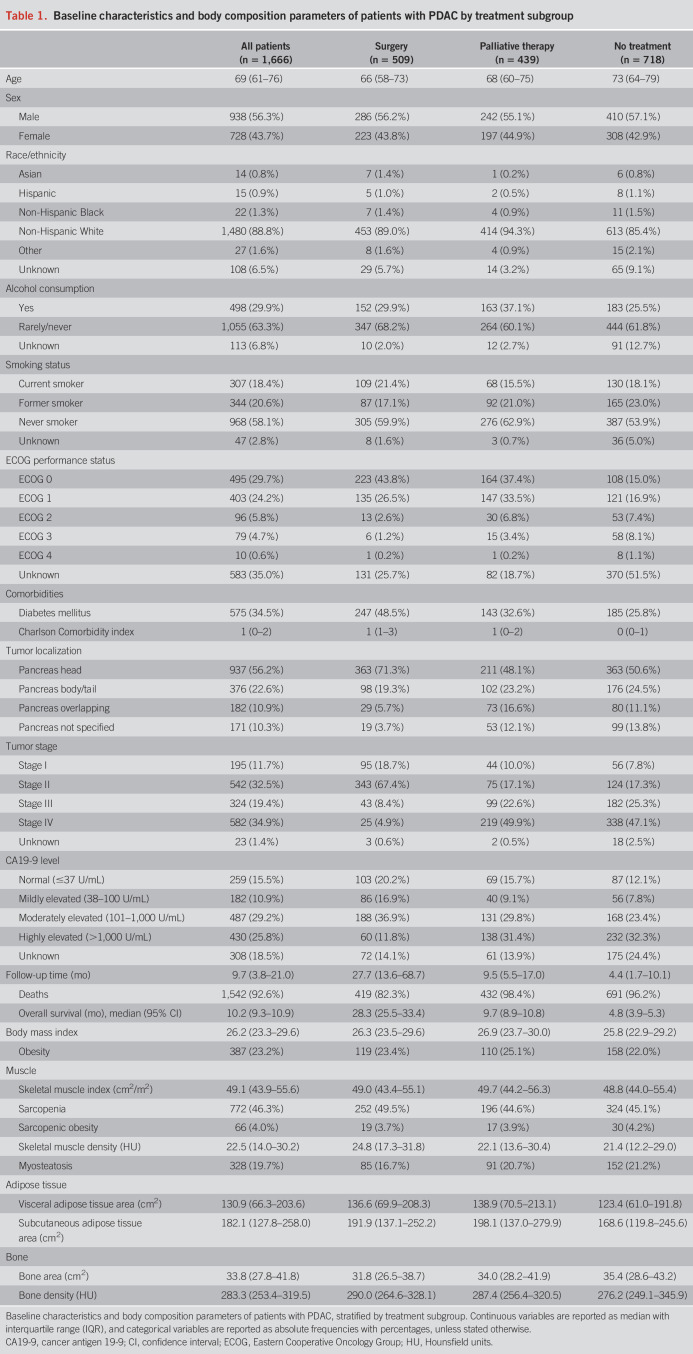
Baseline characteristics and body composition parameters of patients with PDAC by treatment subgroup

### Body composition and OS in surgery subgroup

**Figure 2.**
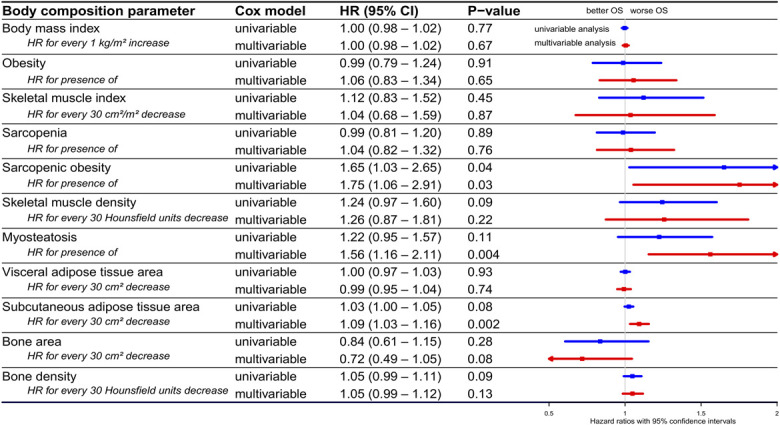
Hazard ratios for mortality in patients with PDAC who underwent surgery. Forest plot showing HRs for mortality and their 95% confidence intervals in patients with PDAC who underwent surgery, based on various body composition parameters, determined using univariable (blue lines) and multivariable (red lines) Cox regression analysis. To study the independent association between various body composition parameters and overall survival, separate multivariable analyses were performed with every body composition parameter individually adjusted for potential confounders (age, sex, race/ethnicity, alcohol consumption, smoking status, ECOG performance status, BMI, comorbidities, diabetes, tumor localization, tumor stage, and CA19-9 level). BMI, body mass index; CA19-9, carbohydrate antigen 19-9; CCI, Charlson Comorbidity index; CI, confidence interval; ECOG, Eastern Cooperative Oncology Group; HR, hazard ratio; OS, overall survival; PDAC, pancreatic ductal adenocarcinoma.

Figure 2 presents the association between body composition parameters and OS in patients with PDAC who underwent surgery. In multivariable analysis, myosteatosis (HR 1.56, 95% CI 1.16–2.11, *P* = 0.004), sarcopenic obesity (HR 1.75, 95% CI 1.06–2.91, *P* = 0.03), and lower SAT (HR 1.09 for every 30 cm^2^ decrease, 95% CI 1.03–1.16, *P* = 0.002) were significantly associated with increased mortality (i.e., worse OS). Kaplan-Meier curves comparing OS based on the presence of sarcopenia, obesity, sarcopenic obesity, and myosteatosis are shown in Supplementary Digital Content (see Supplementary Figure 2, http://links.lww.com/AJG/D845).

### Body composition and OS in palliative therapy subgroup

**Figure 3.**
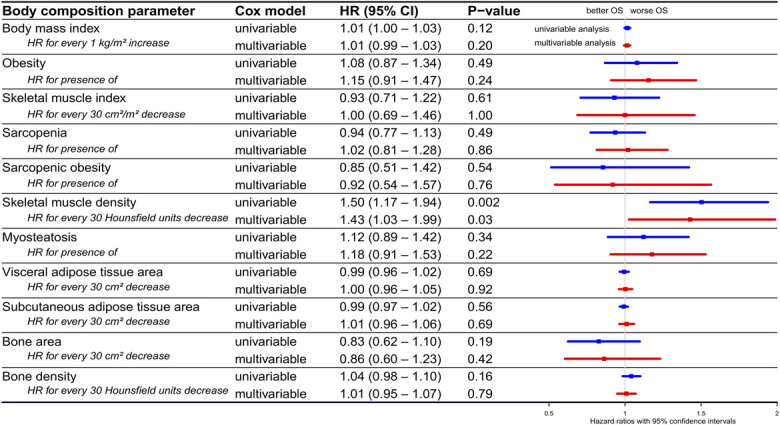
Hazard ratios for mortality in patients with PDAC who received palliative therapy. Forest plot showing HRs for mortality and their 95% confidence intervals in patients with PDAC who received palliative therapy, based on various body composition parameters, determined using univariable (blue lines) and multivariable (red lines) Cox regression analysis. To study the independent association between various body composition parameters and overall survival, separate multivariable analyses were performed with every body composition parameter individually adjusted for potential confounders (age, sex, race/ethnicity, alcohol consumption, smoking status, ECOG performance status, BMI, comorbidities, diabetes, tumor localization, tumor stage, and CA19-9 level). BMI, body mass index; CA19-9, carbohydrate antigen 19-9; CCI, Charlson Comorbidity Index; CI, confidence interval; ECOG, Eastern Cooperative Oncology Group; HR, hazard ratio; OS, overall survival; PDAC, pancreatic ductal adenocarcinoma.

In patients with PDAC who received palliative therapy, lower SMD was significantly associated with worse OS in both univariable analysis (HR 1.50 for every 30 HU decrease, 95% CI 1.17–1.94, *P* = 0.002) and multivariable analysis (HR 1.43 for every 30 HU decrease, 95% CI 1.03–1.99, *P* = 0.03; Figure 3). Kaplan-Meier curves based on the presence of sarcopenia, obesity, sarcopenic obesity, and myosteatosis are provided in Supplementary Digital Content (see Supplementary Figure 3, http://links.lww.com/AJG/D845).

### Body composition and OS in no tumor-targeted treatment subgroup

**Figure 4.**
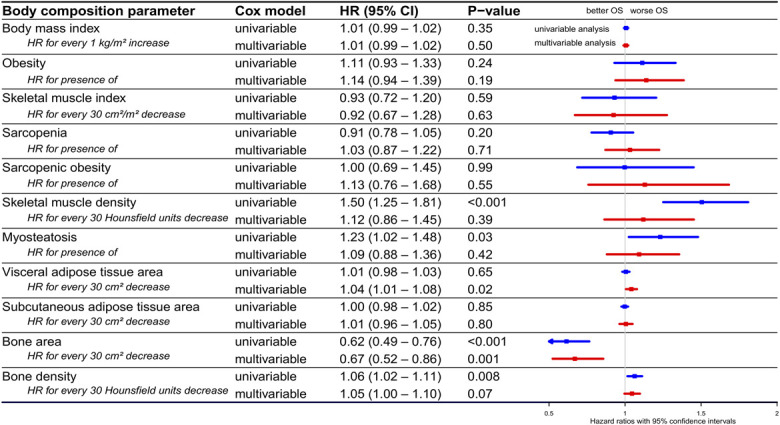
HRs for mortality in patients with PDAC who did not undergo tumor-targeted treatment. Forest plot showing HRs for mortality and their 95% confidence intervals in patients with PDAC who did not undergo tumor-targeted treatment, based on various body composition parameters, determined using univariable (blue lines) and multivariable (red lines) Cox regression analysis. To study the independent association between various body composition parameters and overall survival, separate multivariable analyses were performed with every body composition parameter individually adjusted for potential confounders (age, sex, race/ethnicity, alcohol consumption, smoking status, ECOG performance status, BMI, comorbidities, diabetes, tumor localization, tumor stage, and CA19-9 level). BMI, body mass index; CA19-9, carbohydrate antigen 19-9; CCI, Charlson Comorbidity Index; CI, confidence interval; ECOG, Eastern Cooperative Oncology Group; HR, hazard ratio; OS, overall survival; PDAC, pancreatic ductal adenocarcinoma.

In patients with PDAC who did not undergo tumor-targeted treatment, lower VAT (HR 1.04 for every 30 cm^2^ decrease, 95% CI 1.01–1.08, *P* = 0.02; Figure 4) and greater BA (HR 0.67 for every 30 cm^2^ decrease, 95% CI 0.52–0.86, *P* = 0.001) were independently related to worse OS in multivariable analysis. Kaplan-Meier curves based on the presence of sarcopenia, obesity, sarcopenic obesity, and myosteatosis are shown in Supplementary Digital Content (see Supplementary Figure 4, http://links.lww.com/AJG/D845). An overview of all multivariable analysis results across all 3 subgroups is presented in Supplementary Digital Content (see Supplementary Figure 5, http://links.lww.com/AJG/D845).

## DISCUSSION

This multisite study is the largest to date examining the association between abdominal body composition parameters and OS in various subgroups of patients with PDAC and among the first to apply a deep learning-based 3D approach to routinely performed CT scans. We found that several body composition parameters, such as low SMD, adipose tissue, and sarcopenic obesity, were significantly associated with OS, even after adjusting for potential confounders such as age, ECOG performance status, and tumor stage.

### Myosteatosis

Our most notable finding was that either lower SMD, a continuous variable, or myosteatosis, a dichotomous variable derived from SMD, was independently associated with worse OS in patients who underwent surgery or palliative therapy, which is in line with previous literature (19,21–31). In contrast to these findings, other studies did not find this association (17,18,40–42), highlighting the contradictory nature of current research on body composition in patients with PDAC. This lack of generalizability may be due to several factors, including differences in demographic characteristics and patient cohorts. For instance, most of these studies have been conducted within the Asian population (27,28,42) that has a significantly different body composition compared with the Western population (43). In addition, most studies have either focused solely on patients who underwent surgery (18,28,31) or patients with locally advanced or metastatic disease (7,19,20). Importantly, only a few studies adjusted for comorbidities using the Charlson Comorbidity Index (CCI) (16,23,44), World Health Organization (WHO) Performance Status (30), Eastern Cooperative Oncology Group (ECOG) Performance Status Scale (27), American Society of Anesthesiologists (ASA) Classification (45), or specifically for diabetes mellitus (21,31), despite these being important confounders in the relationship between body composition and OS (46).

Myosteatosis is characterized by excessive fat accumulation within skeletal muscle (47), while skeletal muscle normally contains only minimal amounts of adipose tissue (48). Both skeletal muscle and adipose tissue are considered secretory organs, with myocytes producing myokines and adipocytes producing (proinflammatory) adipokines, which are supposed to maintain a balance (49,50). The increase in adipocytes (producing proinflammatory adipokines) seen in myosteatosis is believed to disrupt this balance, leading to systemic inflammation (51), which may contribute to several negative outcomes such as cachexia, tumor progression, and tumor proliferation (52). Clinical studies in patients with PDAC corroborate this theory, showing that patients with low SMD have lower levels of albumin (19,31) and higher levels of inflammatory blood markers (19), both indicators of systemic inflammation. There are 2 possible explanations for the observed association between myosteatosis and OS. First, myosteatosis may be a manifestation of the systemic effects of PDAC itself, serving as an indicator of disease progression. In this case, myosteatosis reflects the systemic impact of PDAC rather than being an independent driver of worse outcomes. Second, patients who already have myosteatosis—because of factors such as lifestyle, comorbidities, or metabolic dysfunction—may have a predisposition to worse outcomes, independent of PDAC.

### Subcutaneous and visceral adipose tissue

Our study also found that lower SAT (in the surgery subgroup) and lower VAT (in the no tumor-targeted treatment subgroup) were associated with worse OS. Although several smaller studies have investigated the association between SAT and OS in patients with PDAC (18,22,30–33,42,44,53–55), this association has only been observed in a Chinese cohort of patients with irresectable pancreatic cancer (HR 1.31 for high SAT, 95% CI 1.01–1.69, *P* = 0.05 in univariable analysis) (33). Similarly, of all studies examining the association between VAT and OS in patients with PDAC (15,18,20,22,29–33,42,44,45,53–58), only 4 have found a significant association (29,30,32,33). The lack of consistency in these findings may be attributed to the same factors mentioned earlier. A recent study from our group found that both SAT and VAT significantly decreased over time in CT scans up to 36 months before PDAC diagnosis (59). This finding suggests that patients with low SAT and VAT at the moment of diagnosis may have experienced more adipose tissue loss before diagnosis, possibly reflecting a more advanced disease stage that leads to worse OS.

### Sarcopenia and sarcopenic obesity

Interestingly, we did not observe an association between low SMI (i.e., sarcopenia) and OS in our cohort, despite several meta-analyses linking sarcopenia to worse OS in patients with PDAC, and similar sarcopenia rates in our study (9–14). However, the combined presence of sarcopenia and obesity (i.e., sarcopenic obesity) was associated with worse OS in the subgroup of patients with PDAC who underwent surgery. This finding is consistent with previous research (7,10,15–20,29), although other studies did not observe this association (23,30,54,57). Chronic systemic inflammation caused by obesity might contribute to worse OS (60), similar to the inflammation caused by myosteatosis. Furthermore, obesity has been linked with major complications after pancreatic surgery (61,62), which could explain why sarcopenic obesity was associated with worse OS specifically in the surgery subgroup.

### Bone area

In addition, we found that greater BA was strongly associated with worse OS in the subgroup of patients who did not undergo tumor-targeted treatment, which is an unexpected finding that remains unexplained. This observation seems to contradict our previous research, which used the same body composition segmentation algorithm on the same cohort of patients to study prediagnostic changes and found a 10% decrease in BA in the 6 months before PDAC diagnosis (59). According to this trend, one would expect that patients with lower BA at the time of diagnosis have more advanced disease and therefore worse OS, rather than patients with greater BA. While bone metastases might explain this finding, they occur in only about 10% of patients with PDAC and typically lead to altered bone density rather than increased BA (63). Although this finding could be due to chance, the observed association was relatively strong (*P* < 0.001 in univariable and *P* = 0.001 in multivariable analysis), with a notably higher HR compared with other treatment groups, suggesting that this finding may not be entirely random. BA showed only a weak Pearson correlation (r < 0.1) with other body composition parameters that were significantly associated with OS, indicating that the observed association is unlikely to be driven by correlations with another body composition parameter. To better understand this phenomenon, it would be valuable to explore not only the temporal changes in BA before diagnosis but also how BA evolves after diagnosis as the disease progresses.

### Strengths

Our study has several strengths, including its large sample size, extensive adjustment for potential confounders (such as ECOG performance status, comorbidities, clinical factors, and CA19-9), and comprehensive body composition assessment. Only a recent study by Keyl et al. used a similar deep learning-based algorithm with a 3-dimensional approach in patients with PDAC, but focused solely on muscle tissue in patients with advanced disease (25). While our method of measuring body composition across a 20 cm vertical section of the abdomen is more comprehensive than estimating body composition from a single CT slice at the L3 level, most previous literature on body composition is based on single-slice assessment, limiting comparability with novel methods like ours. Furthermore, the established cut-off values for myosteatosis and sarcopenia are based on SMD and SMI at L3 (64,65). Because SMD and SMI vary across the abdomen, these cut-off values are less applicable to our body composition measurement approach (66). To overcome this challenge, we used the reference ranges reported by Weston et al (37) who applied the same abdominal segmentation algorithm to characterize CT-based body composition in a population-representative American cohort. For future studies using a similar measurement approach as ours, we advocate for using the same reference ranges to ensure consistency and comparability.

### Limitations

Next to the limited comparability with older research, our study has several other limitations. First, owing to its retrospective design, selection bias cannot be entirely ruled out. Our cohort primarily consisted of non-Hispanic White patients, which may limit the generalizability of our findings to other populations. Furthermore, although we stratified our cohort by treatment type, we did not account for detailed treatment information such as type of surgery or chemotherapy regimens. Despite being the largest study to date, further subdividing our cohort could lead to smaller subgroups with insufficient statistical power. In addition, while we assessed morphological muscle parameters (SMI and SMD) using CT scans, we could not evaluate actual muscle quality or strength through clinical tests because of the study's retrospective nature. Although imaging-based muscle quality relates to actual strength (67), clinical tests could provide more precise insights.

### Future directions and clinical implications

A promising next step after this study would be to develop and validate a prediction model for PDAC prognosis at the time of diagnosis. Such a model could integrate demographic and clinical information, tumor characteristics, laboratory results, and CT-based body composition parameters. This could aid in risk stratification, personalized treatment planning, and early identification of patients who may benefit from more or less aggressive treatment. In addition, further investigation into how body composition parameters evolve after PDAC diagnosis, and their relationship to outcomes such as chemotherapy response and toxicity, could deepen our understanding of the role of body composition in PDAC. Prospective studies combining nutritional, exercise, and metabolic interventions are warranted to determine whether improving muscle quality or mitigating myosteatosis translates into better tolerance to therapy and survival (68,69). Although small-scale studies suggest that such programs may improve SMI and SMD, their impact on survival outcomes remains to be established (70,71).

### Conclusion

In conclusion, our study found that body composition parameters, such as low SMD, adipose tissue, and sarcopenic obesity, are independently associated with worse OS across various subgroups of patients with PDAC. Given that CT imaging is routinely performed for diagnostic and staging purposes, these parameters can be readily extracted using a deep learning-based algorithm. Future research should explore the clinical implications of these body composition parameters for treatment planning.

## CONFLICTS OF INTEREST

**Guarantors of the article:** Koen J.H. Wijsman, MD, MSc; Yan Bi, MD, PhD.

**Specific author contributions:** K.J.H.W., D.C.F.K., M.B.W., Y.B., J.E.v.H., M.E.v.L., A.D.W.: conceptualization. K.J.H.W., D.C.F.K., A.D.W.: data curation. K.J.H.W., A.D.W.: formal analysis. K.J.H.W., M.B.W., Y.B., J.E.v.H., M.E.v.L.: funding acquisition. K.J.H.W.: investigation. K.J.H.W., D.C.F.K., M.B.W., Y.B., J.E.v.H., M.E.v.L., A.D.W., B.R.G.: methodology. K.J.H.W.: project administration. M.B.W., Y.B.: resources. K.J.H.W., A.D.W.: software. D.C.F.K., M.B.W., Y.B., J.E.v.H., M.E.v.L.: supervision. K.J.H.W., B.R.G.: validation. K.J.H.W.: visualization. K.J.H.W.: writing—original draft. D.C.F.K., M.B.W., Y.B., J.E.v.H., M.E.v.L., A.D.W., B.R.G., A.M.B., H.M.B., J.S.D.M.: writing—review and editing.

**Financial support:** K.J.H.W.: received grants from the René Vogels Foundation, Nijbakker-Morra Foundation, Prof. dr. A.E. Meinders Fund, LUF International Study Fund, Professor Chris Gips Foundation, Minerva Scholarship Fund, CAPER Travel Grant, Quintus Fund, LUSTRA+ Scholarship, Leiden University Trustee Funds, ESC Travel Grant, KNMG Dick Held, and Hanarth Foundation. The funding sources had no involvement in the study design; collection, analysis, and interpretation of data; writing of the report; or decision to submit the article for publication. The research was conducted independently by the authors.

**Potential competing interests:** J.E.v.H. reported receiving lecture fees from Cook Medical, Boston Scientific, Fujifilm, and Falk, as well as a consultancy fee from Olympus Medical, all outside the submitted work. M.B.W. reported consulting for Boston Scientific, CDX Diagnostics, ClearNote Health, Cosmo Pharmaceuticals, Digma Medical, Endostart, Endiatix, Fujifilm, Medtronic, Surgical Automations, Ohelio Ltd, Venn Bioscience; research grants from Fujifilm, Boston Scientific, Olympus, Medtronic, Cosmo Intelligent Medical Devices; stock/stock options in Virgo Inc., Surgical Automation; consulting on behalf of Mayo Clinic for Boston Scientific, Microtek; all outside the submitted work. All other authors report no potential conflicts of interest.

**Study Highlights**

**WHAT IS KNOWN**

✓ Pancreatic ductal adenocarcinoma (PDAC) has a poor prognosis, with a 5-year survival rate of only 5%.
✓ A better understanding of the factors influencing prognosis in PDAC could support more personalized treatment approaches.
✓ Cancer prognosis is influenced by both tumor-related and patient-related factors, including body composition.
✓ Computed tomography-derived body composition parameters have been associated with survival outcomes in various cancers.
✓ Previous studies on computed tomography-based body composition and survival in patients with PDAC have reported inconsistent results.

**WHAT IS NEW HERE**

✓ This multisite study is the largest to date investigating the association between body composition parameters and overall survival in patients with PDAC.
✓ It is among the first to apply a deep learning-based 3D approach to quantify body composition in this patient population.
✓ Several body composition parameters, such as low skeletal muscle density, adipose tissue, and sarcopenic obesity, were significantly associated with overall survival, even after adjustment for potential confounders.
✓ Evaluating body composition at the time of diagnosis could enhance clinical decision-making and enable more personalized treatment strategies.

## Supplementary Material

## References

[1] Siegel RL Giaquinto AN Jemal A. Cancer statistics, 2024. CA Cancer J Clin 2024;74(1):12–49. 38230766 10.3322/caac.21820

[2] Rahib L Wehner MR Matrisian LM . Estimated projection of US cancer incidence and death to 2040. JAMA Netw Open 2021;4(4):e214708. 33825840 10.1001/jamanetworkopen.2021.4708 PMC8027914

[3] Kokkinakis S Kritsotakis EI Maliotis N . Complications of modern pancreaticoduodenectomy: A systematic review and meta-analysis. Hepatobiliary Pancreat Dis Int 2022;21(6):527–37. 35513962 10.1016/j.hbpd.2022.04.006

[4] Groot VP Rezaee N Wu W . Patterns, timing, and predictors of recurrence following pancreatectomy for pancreatic ductal adenocarcinoma. Ann Surg 2018;267(5):936–45. 28338509 10.1097/SLA.0000000000002234

[5] Park W Chawla A O’Reilly EM. Pancreatic cancer: A review. JAMA. 2021;326(9):851–62. 34547082 10.1001/jama.2021.13027 PMC9363152

[6] Mackay TM Latenstein AE Augustinus S . Implementation of best practices in pancreatic cancer care in the Netherlands: A stepped-wedge randomized clinical trial. JAMA Surg 2024;159(4):429–37. 38353966 10.1001/jamasurg.2023.7872 PMC10867778

[7] Dalal S Hui D Bidaut L . Relationships among body mass index, longitudinal body composition alterations, and survival in patients with locally advanced pancreatic cancer receiving chemoradiation: A pilot study. J Pain Symptom Manage 2012;44(2):181–91. 22695045 10.1016/j.jpainsymman.2011.09.010 PMC3990439

[8] Borga M West J Bell JD . Advanced body composition assessment: From body mass index to body composition profiling. J Invest Med 2018;66(5):1–9. 10.1136/jim-2018-000722 PMC5992366 29581385

[9] Liu C An L Zhang S . Association between preoperative sarcopenia and prognosis of pancreatic cancer after curative-intent surgery: A updated systematic review and meta-analysis. World J Surg Oncol 2024;22(1):38. 38287345 10.1186/s12957-024-03310-y PMC10825983

[10] Mintziras I Miligkos M Wächter S . Sarcopenia and sarcopenic obesity are significantly associated with poorer overall survival in patients with pancreatic cancer: Systematic review and meta-analysis. Int J Surg 2018;59:19–26. 30266663 10.1016/j.ijsu.2018.09.014

[11] Pierobon ES Moletta L Zampieri S . The prognostic value of low muscle mass in pancreatic cancer patients: A systematic review and meta-analysis. J Clin Med 2021;10(14):3033. 34300199 10.3390/jcm10143033 PMC8306134

[12] Thormann M Hinnerichs M Barajas Ordonez F . Sarcopenia is an independent prognostic factor in patients with pancreatic cancer—A meta-analysis. Acad Radiol 2023;30(8):1552–61. 36564257 10.1016/j.acra.2022.10.025

[13] Yang L Liao X Xie Z . Prognostic value of pretreatment skeletal muscle index in pancreatic carcinoma patients: A meta-analysis. Medicine 2023;102(19):e33663. 37171343 10.1097/MD.0000000000033663 PMC10174348

[14] Zhong L Liu J Xia M . Effect of sarcopenia on survival in patients after pancreatic surgery: A systematic review and meta-analysis. Systematic review. Front Nutr 2024;10:1315097. 38260056 10.3389/fnut.2023.1315097 PMC10800600

[15] Cooper AB Slack R Fogelman D . Characterization of anthropometric changes that occur during neoadjuvant therapy for potentially resectable pancreatic cancer. Ann Surg Oncol 2015;22(7):2416–23. 25519927 10.1245/s10434-014-4285-2 PMC11831732

[16] Gruber ES Jomrich G Tamandl D . Sarcopenia and sarcopenic obesity are independent adverse prognostic factors in resectable pancreatic ductal adenocarcinoma. PLoS One 2019;14(5):e0215915. 31059520 10.1371/journal.pone.0215915 PMC6502449

[17] Kays JK Shahda S Stanley M . Three cachexia phenotypes and the impact of fat-only loss on survival in FOLFIRINOX therapy for pancreatic cancer. J Cachexia Sarcopenia Muscle 2018;9(4):673–84. 29978562 10.1002/jcsm.12307 PMC6104116

[18] Peng Y-C Wu C-H Tien Y-W . Preoperative sarcopenia is associated with poor overall survival in pancreatic cancer patients following pancreaticoduodenectomy. Eur Radiol 2021;31(4):2472–81. 32974690 10.1007/s00330-020-07294-7

[19] Rollins KE Tewari N Ackner A . The impact of sarcopenia and myosteatosis on outcomes of unresectable pancreatic cancer or distal cholangiocarcinoma. Clin Nutr 2016;35(5):1103–9. 26411749 10.1016/j.clnu.2015.08.005

[20] Tan BHL Birdsell LA Martin L . Sarcopenia in an overweight or Obese patient is an adverse prognostic factor in pancreatic cancer. Clin Cancer Res 2009;15(22):6973–9. 19887488 10.1158/1078-0432.CCR-09-1525

[21] Bi S Jiang Y Guan G . Prognostic value of myosteatosis and creatinine-to-cystatin C ratio in patients with pancreatic cancer who underwent radical surgery. Ann Surg Oncol 2024;31(5):2913–24. 38319516 10.1245/s10434-024-14969-8

[22] Cai Z-W Li J-L Liu M . Low preoperative skeletal muscle index increases the risk of mortality among resectable pancreatic cancer patients: A retrospective study. World J Gastrointest Surg 2022;14(12):1350–62. 36632124 10.4240/wjgs.v14.i12.1350 PMC9827571

[23] Damm M Efremov L Jalal M . Body composition parameters predict survival in pancreatic cancer—A retrospective multicenter analysis. United Eur Gastroenterol J 2023;11(10):998–1009. 10.1002/ueg2.12489 PMC10720684 37987099

[24] Griffin OM Duggan SN Ryan R . Characterising the impact of body composition change during neoadjuvant chemotherapy for pancreatic cancer. Pancreatology 2019;19(6):850–7. 31362865 10.1016/j.pan.2019.07.039

[25] Keyl J Bucher A Jungmann F . Prognostic value of deep learning-derived body composition in advanced pancreatic cancer—A retrospective multicenter study. ESMO Open 2024;9(1):102219. 38194881 10.1016/j.esmoop.2023.102219 PMC10837775

[26] Kim DW Ahn H Kim KW Prognostic value of sarcopenia and myosteatosis in patients with resectable pancreatic ductal adenocarcinoma. Korean J Radiol. 2022;23(11):1055–66. 36098341 10.3348/kjr.2022.0277 PMC9614291

[27] Kim I-H Choi MH Lee IS . Clinical significance of skeletal muscle density and sarcopenia in patients with pancreatic cancer undergoing first-line chemotherapy: A retrospective observational study. BMC Cancer 2021;21(1):77. 33461517 10.1186/s12885-020-07753-w PMC7814715

[28] Okumura S Kaido T Hamaguchi Y . Impact of preoperative quality as well as quantity of skeletal muscle on survival after resection of pancreatic cancer. Surgery 2015;157(6):1088–98. 25799468 10.1016/j.surg.2015.02.002

[29] Okumura S Kaido T Hamaguchi Y . Visceral adiposity and sarcopenic visceral obesity are associated with poor prognosis after resection of pancreatic cancer. Ann Surg Oncol 2017;24(12):3732–40. 28871520 10.1245/s10434-017-6077-y

[30] Sohal DPS Boutin RD Lenchik L . Body composition measurements and clinical outcomes in patients with resectable pancreatic adenocarcinoma—Analysis from SWOG S1505. J Gastrointest Surg 2024;28(3):232–5. 38445914 10.1016/j.gassur.2023.12.022 PMC13270577

[31] van Dijk DP Bakens MJ Coolsen MME . Low skeletal muscle radiation attenuation and visceral adiposity are associated with overall survival and surgical site infections in patients with pancreatic cancer. J Cachexia Sarcopenia Muscle 2017;8(2):317–26. 27897432 10.1002/jcsm.12155 PMC5377384

[32] Beetz NL Geisel D Maier C . Influence of baseline CT body composition parameters on survival in patients with pancreatic adenocarcinoma. J Clin Med 2022;11(9):2356. 35566483 10.3390/jcm11092356 PMC9105849

[33] Bian X Dai H Feng J . Prognostic values of abdominal body compositions on survival in advanced pancreatic cancer. Medicine 2018;97(22):e10988. 29851855 10.1097/MD.0000000000010988 PMC6393092

[34] Winder C Clark M Frood R . Automated extraction of body composition metrics from abdominal CT or MR imaging: A scoping review. Eur J Radiol 2024;181:111764. 39368243 10.1016/j.ejrad.2024.111764

[35] Elm Ev Altman DG Egger M . Strengthening the reporting of observational studies in epidemiology (STROBE) statement: Guidelines for reporting observational studies. BMJ 2007;335(7624):806–8. 17947786 10.1136/bmj.39335.541782.AD PMC2034723

[36] Weston AD Korfiatis P Kline TL . Automated abdominal segmentation of CT scans for body composition analysis using deep learning. Radiology 2019;290(3):669–79. 30526356 10.1148/radiol.2018181432

[37] Weston AD Grossardt BR Garner HW . Abdominal body composition reference ranges and association with chronic conditions in an age- and sex-stratified representative sample of a geographically defined American population. J Gerontol A Biol Sci Med Sci 2024;79(4):glae055. 38373180 10.1093/gerona/glae055 PMC10949446

[38] Franco Valle K Lubner MG Pickhardt PJ. Computed tomography assessment of sarcopenic myosteatosis for predicting overall survival in colorectal carcinoma: Systematic review. J Comput Assist Tomogr 2022;46(2):157–62. 35297571 10.1097/RCT.0000000000001281

[39] Zamboni M Mazzali G Fantin F . Sarcopenic obesity: A new category of obesity in the elderly. Nutr Metab Cardiovasc Dis 2008;18(5):388–95. 18395429 10.1016/j.numecd.2007.10.002

[40] Akahori T Sho M Kinoshita S . Prognostic significance of muscle attenuation in pancreatic cancer patients treated with neoadjuvant chemoradiotherapy. World J Surg 2015;39(12):2975–82. 26296840 10.1007/s00268-015-3205-3

[41] BarrÈre APN Piovacari SMF UsÓn Junior PLS . Body composition impact on survival and toxicity of treatment in pancreatic cancer: Cross-sectional pilot study. Arquivos de Gastroenterologia 2020;57(3):278–82. 33027479 10.1590/S0004-2803.202000000-52

[42] Choi MH Yoon SB Lee K . Preoperative sarcopenia and post‐operative accelerated muscle loss negatively impact survival after resection of pancreatic cancer. J Cachexia Sarcopenia Muscle 2018;9(2):326–34. 29399990 10.1002/jcsm.12274 PMC5879976

[43] Deurenberg P Yap M Van Staveren WA. Body mass index and percent body fat: A meta analysis among different ethnic groups. Int J Obes 1998;22(12):1164–71. 10.1038/sj.ijo.0800741 9877251

[44] Sugimoto M Farnell MB Nagorney DM . Decreased skeletal muscle volume is a predictive factor for poorer survival in patients undergoing surgical resection for pancreatic ductal adenocarcinoma. J Gastrointest Surg 2018;22(5):831–9. 29392613 10.1007/s11605-018-3695-z PMC6057620

[45] Gaujoux S Torres J Olson S . Impact of obesity and body fat distribution on survival after pancreaticoduodenectomy for pancreatic adenocarcinoma. Ann Surg Oncol 2012;19(9):2908–16. 22411205 10.1245/s10434-012-2301-y

[46] Kim S Leng XI Kritchevsky SB. Body composition and physical function in older adults with various comorbidities. Innov Aging 2017;1(1):igx008. 30480107 10.1093/geroni/igx008 PMC6177091

[47] Ahn H Kim DW Ko Y . Updated systematic review and meta-analysis on diagnostic issues and the prognostic impact of myosteatosis: A new paradigm beyond sarcopenia. Ageing Res Rev 2021;70:101398. 34214642 10.1016/j.arr.2021.101398

[48] Lexell J. Human aging, muscle mass, and fiber type composition. J Gerontol A Biol Sci Med Sci 1995;50(Spec No):11–6. 7493202 10.1093/gerona/50a.special_issue.11

[49] Lutz CT Quinn LS. Sarcopenia, obesity, and natural killer cell immune senescence in aging: Altered cytokine levels as a common mechanism. Aging (Albany NY) 2012;4(8):535–46. 22935594 10.18632/aging.100482 PMC3461341

[50] Pedersen BK Febbraio MA. Muscles, exercise and obesity: Skeletal muscle as a secretory organ. Nat Rev Endocrinol 2012;8(8):457–65. 22473333 10.1038/nrendo.2012.49

[51] Malietzis G Aziz O Jenkins JT . Low muscularity and myosteatosis is related to the host systemic inflammatory response in patients undergoing surgery for colorectal cancer. Ann Surg 2016;263(6):e81. 25643288 10.1097/SLA.0000000000001113

[52] Coussens LM Werb Z. Inflammation and cancer. Nature 2002;420(6917):860–7. 12490959 10.1038/nature01322 PMC2803035

[53] Cloyd JM Nogueras-González GM Prakash LR . Anthropometric changes in patients with pancreatic cancer undergoing preoperative therapy and pancreatoduodenectomy. J Gastrointest Surg 2018;22(4):703–12. 29230694 10.1007/s11605-017-3618-4 PMC6022283

[54] Danai LV Babic A Rosenthal MH . Altered exocrine function can drive adipose wasting in early pancreatic cancer. Nature 2018;558(7711):600–4. 29925948 10.1038/s41586-018-0235-7 PMC6112987

[55] Takaichi S Tomimaru Y Kobayashi S . Change impact of body composition during neoadjuvant chemoradiotherapy in patients with resectable and borderline resectable pancreatic ductal adenocarcinoma undergoing pancreatectomy. Ann Surg Oncol 2023;30(4):2458–68. 36575288 10.1245/s10434-022-12985-0

[56] Hsu T-MH Schawkat K Berkowitz SJ . Artificial intelligence to assess body composition on routine abdominal CT scans and predict mortality in pancreatic cancer–A recipe for your local application. Eur J Radiol 2021;142:109834. 34252866 10.1016/j.ejrad.2021.109834

[57] Naumann P Eberlein J Farnia B . Cachectic body composition and inflammatory markers portend a poor prognosis in patients with locally advanced pancreatic cancer treated with chemoradiation. Cancers 2019;11(11):1655. 31717736 10.3390/cancers11111655 PMC6895786

[58] Ninomiya G Fujii T Yamada S . Clinical impact of sarcopenia on prognosis in pancreatic ductal adenocarcinoma: A retrospective cohort study. Int J Surg 2017;39:45–51. 28110029 10.1016/j.ijsu.2017.01.075

[59] Klatte DCF Weston A Ma Y . Temporal trends in body composition and metabolic markers prior to diagnosis of pancreatic ductal adenocarcinoma. Clin Gastroenterol Hepatol 2024;22(9):1830–8.e9. 38703880 10.1016/j.cgh.2024.03.038

[60] Deng T Lyon CJ Bergin S . Obesity, inflammation, and cancer. Annu Rev Pathol Mech Dis 2016;11:421–49. 10.1146/annurev-pathol-012615-044359 27193454

[61] House MG Fong Y Arnaoutakis DJ . Preoperative predictors for complications after pancreaticoduodenectomy: Impact of BMI and body fat distribution. J Gastrointest Surg 2008;12(2):270–8. 18060467 10.1007/s11605-007-0421-7

[62] Lucassen CJ Groen JV Aziz MH . Visceral adipose tissue is a better predictor than BMI in the alternative Fistula Risk Score in patients undergoing pancreatoduodenectomy. HPB (Oxford) 2022;24(10):1679–87. 35527105 10.1016/j.hpb.2022.03.004

[63] Puri A Chang J Tanner N . Skeletal metastases in advanced pancreatic ductal adenocarcinoma: A retrospective analysis. J Gastrointest Oncol 2021;12(2):455–63. 34012639 10.21037/jgo-20-361 PMC8107595

[64] Martin L Birdsell L MacDonald N . Cancer cachexia in the age of obesity: Skeletal muscle depletion is a powerful prognostic factor, independent of body mass index. J Clin Oncol 2013;31(12):1539–47. 23530101 10.1200/JCO.2012.45.2722

[65] Prado CMM Lieffers JR McCargar LJ . Prevalence and clinical implications of sarcopenic obesity in patients with solid tumours of the respiratory and gastrointestinal tracts: A population-based study. Lancet Oncol 2008;9(7):629–35. 18539529 10.1016/S1470-2045(08)70153-0

[66] Derstine BA Holcombe SA Ross BE . Skeletal muscle cutoff values for sarcopenia diagnosis using T10 to L5 measurements in a healthy US population. Sci Rep 2018;8(1):11369. 30054580 10.1038/s41598-018-29825-5 PMC6063941

[67] Delmonico MJ Harris TB Visser M . Longitudinal study of muscle strength, quality, and adipose tissue infiltration. Am J Clin Nutr 2009;90(6):1579–85. 19864405 10.3945/ajcn.2009.28047 PMC2777469

[68] Rovesti G Valoriani F Rimini M . Clinical implications of malnutrition in the management of patients with pancreatic cancer: Introducing the concept of the nutritional oncology board. Nutrients 2021;13(10):3522. 34684523 10.3390/nu13103522 PMC8537095

[69] Prado CM Landi F Chew STH . Advances in muscle health and nutrition: A toolkit for healthcare professionals. Clin Nutr 2022;41(10):2244–63. 36081299 10.1016/j.clnu.2022.07.041

[70] Parker NH Gorzelitz J Ngo-Huang A . The role of home-based exercise in maintaining skeletal muscle during preoperative pancreatic cancer treatment. Integr Cancer Therapies 2021;20:1534735420986615. 10.1177/1534735420986615 PMC8056559 33870744

[71] Mikkelsen MK Lund CM Vinther A . Effects of a 12-Week multimodal exercise intervention among older patients with advanced cancer: Results from a randomized controlled trial. Oncologist 2022;27(1):67–78. 34498352 10.1002/onco.13970 PMC8842365

