# Supplementary material for: Association Between Computed Tomography–Based AI-Derived Body Composition and Survival in Patients With Pancreatic Ductal Adenocarcinoma
Source: Am J Gastroenterol. 2025 Dec 22;121(4):982–92. doi: 10.14309/ajg.0000000000003896 (PMC13038073; doi:10.14309/ajg.0000000000003896)
Supplement: Supplementary file 2 [file acg-121-982-s002.pdf]

ONLINE SUPPLEMENTAL MATERIALS

Title

Association Between CT-Based AI-Derived Body Composition and Survival in Patients With Pancreatic Ductal Adenocarcinoma

Authors

Koen J.H. Wijsman, MD, MSc[1,2], Derk C.F. Klatte, MD, PhD[1,2]; Hani M. Babiker, MD[3]; Aleksander M. Bogdanski, BSc[1,2]; Brandon R. Grossardt, MS[4]; Jeanin E. van Hooft, MD, PhD, MBA[1]; Monique E. van Leerdam, MD, PhD, MSc[1,5], J. Sven D. Mieog, MD, PhD[6]; Alexander D. Weston, PhD[7]; Michael B. Wallace, MD, MPH[2]; Yan Bi, MD, PhD[2]

Affiliations

[1] Department of Gastroenterology and Hepatology, Leiden University Medical Center, Leiden, the Netherlands

[2] Division of Gastroenterology and Hepatology, Department of Medicine, Mayo Clinic, Jacksonville, FL, USA

[3] Division of Hematology and Oncology, Department of Medicine, Mayo Clinic, Jacksonville, FL, USA

[4] Department of Quantitative Health Sciences, Mayo Clinic, Rochester, MN, USA

[5] Department of Gastrointestinal Oncology, Netherlands Cancer Institute, Amsterdam, the Netherlands

[6] Department of Surgery, Leiden University Medical Center, Leiden, the Netherlands

[7] Department of Quantitative Health Sciences, Mayo Clinic, Jacksonville, FL, USA

Corresponding Author

Koen J.H. Wijsman, MD, MSc (email: K.J.H.Wijsman@lumc.nl)

Department of Gastroenterology and Hepatology – Leiden University Medical Center

ONLINE SUPPLEMENTAL MATERIALS

Legend

- **Supplemental Methods**
- **Supplemental Table 1**: Age- and Sex-Specific Cut-Off Values for the Presence of Sarcopenia and Myosteatosis
- **Supplemental Table 2**: Age- and Sex-Specific Distribution of Body Composition Parameters
- **Supplemental Table 3**: Breakdown of the Charlson Comorbidity Index (CCI) for All PDAC Patients and Treatment Subgroups
- **Supplemental Table 4**: Detailed Treatment Characteristics of Patients with PDAC
- **Supplemental Table 5**: Imaging Acquisition and Reconstruction Parameters
- **Supplemental Figure 1**: Kaplan-Meier Curve for Overall Survival of Patients with PDAC Stratified by Treatment Subgroups
- **Supplemental Figure 2**: Kaplan-Meier Curves of Patients with PDAC Who Underwent Surgery
- **Supplemental Figure 3**: Kaplan-Meier Curves of Patients with PDAC Who Received Palliative Therapy
- **Supplemental Figure 4**: Kaplan-Meier Curves of Patients with PDAC Who Did Not Undergo Tumor-Targeted Treatment

SUPPLEMENTAL METHODS

Patient Selection and Data Collection

Patients diagnosed with PDAC from 2000 to 2020 at Mayo Clinic sites in Minnesota, Florida, and Arizona were identified using International Classification of Diseases for Oncology (ICD-O) codes C250-C254 and C257-C259.

Demographic data, including sex, race, and ethnicity, were collected from the patient medical records. Although we attempted to specify each patient's race and ethnicity, 27 patients were recorded as 'other' in the medical records, and their specific race or ethnicity could not be determined. As a result, these patients were included in a separate group labeled 'other'. Data on alcohol consumption, smoking status, and Eastern Cooperative Oncology Group (ECOG) Performance Status were extracted from patient medical records. Pre-existing comorbidities were assessed using International Classification of Diseases (ICD-9 and ICD-10) codes and were aggregated into a modified version of the Charlson Comorbidity Index (CCI), with age, diabetes, and tumor stage excluded, as these factors were separately accounted for in the regression analyses to adjust for confounding.

Tumor data included localization and staging, which was conducted according to the American Joint Committee on Cancer (AJCC) staging system in effect at the time of diagnosis (Fifth through Eighth edition for this study). For patients who underwent surgery, we used the pathological TNM (pTNM) staging, whereas for all other patients, the clinical TNM (cTNM) staging was used. Cancer antigen (CA)19-9 levels were obtained from patient medical records and categorized as normal (≤ 37 U/mL), mildly elevated (38–100 U/mL), moderately elevated (101–1000 U/mL), and highly elevated (> 1000 U/mL).

Treatment data included information on surgery, radiation therapy, and systemic therapy such as chemotherapy. Survival data included the date of death or the date of last patient contact. Patients were followed up until 31 July 2025.

Measurement of Body Composition Parameters

Body composition parameters were extracted from diagnostic CT scans using a previously validated deep learning-based algorithm, which employs a three-dimensional U-Net Convolutional Neural Network (CNN) model that achieved a Dice similarity coefficient of 0.92-0.98 (**Figure 1**).[35] The algorithm enforces that the scan must be an axial reconstruction with at least 20 slices and resamples all 3D images to 512×512×128 voxels using linear interpolation. For a standard abdomen CT, this corresponds to approximately 2-3 mm slice thickness, though this may be closer to 5 mm or more for chest-abdomen-pelvis scans. The algorithm automatically selects a 20 cm vertical section of the abdomen, centered at the midpoint of the third lumbar vertebra (L3), and segments every axial CT slice within this 20 abdominal section into skeletal muscle area (SMA, cm^2), visceral adipose tissue area (VAT, cm^2), subcutaneous adipose tissue area (SAT, cm^2), and bone area (BA, cm^2). For each tissue type, the values in all segmented CT slices are summed to obtain 3D volumetric measurements. Subsequently, the 3D measurements are divided by the number of CT slices multiplied by the slice thickness to derive 2D area measurements (cm^2). These 2D measurements (cm^2 instead of cm^3) align best with standard reporting of body composition parameters in comparable literature, which typically uses single-slice segmentation at the level of the third lumbar vertebra (L3). To account for differences in stature, skeletal muscle area was indexed to the patient's height in meters squared, resulting in the skeletal muscle index (SMI, cm^2/m^2).

Skeletal muscle density (SMD) and bone density (BD) were defined as the average Hounsfield unit (HU) attenuation of all voxels within their respective compartments. The Hounsfield scale, utilized in CT imaging, measures the radiodensity of tissues within the body, with lower HU values indicating lower tissue density. The term "density" was used instead of "attenuation" to provide a more intuitive concept for clinical readers.

Failure cases occurred for several reasons, including duplicate SeriesUIDs, mislabeling of non-abdomen scans (typically chest scans), low-dose CT acquisitions, non-primary acquisitions such as Maximum Intensity Projections (MIPs), metal artifacts, patients with arms positioned down, or patients scanned lying on their side or stomach. To ensure accuracy, all algorithm outputs were manually reviewed for each series, and cases with errors were excluded from the analysis.

Deep learning model inference was performed using the Tensorflow package version 2.6.024 in Python 3.7.12. GPU acceleration was used for model inference using 4 NVIDIA T4 GPUs (CUDA version 11.2) (NVIDIA Corp, Santa Clara, CA). Model inference was performed using the VertexAI feature of Google Cloud Platform (Google Cloud Platform, Mountain View, CA). All abdominal segmentations generated by the body composition algorithm were manually reviewed by an expert (A.D.W.) to ensure accurate segmentation. Detailed information on the training and validation of the algorithm can be found in a separate publication.[35]

Definitions of Obesity, Sarcopenia, Sarcopenic Obesity and Myosteatosis

Weight data (missing for 174 patients, 10.4%) were imputed using Multivariate Imputation by Chained Equations (MICE). As age, sex, height, and body composition parameters (including total abdominal area, SMI, SMD, SAT, VAT, BA, and BD) were available for these patients, the imputations are expected to be sufficiently accurate.

Cut-off values for the presence of sarcopenia and myosteatosis were based on a previous study that used the same abdominal segmentation algorithm to report reference ranges for CT-based body composition parameters in a population-representative geographically defined American cohort.[36] A population-representative cohort reflects the characteristics of the general population, including variations in age, health status, and other demographic factors. This ensures that reference ranges for body composition are not biased by selective cohorts, such as patients with specific medical conditions or exceptionally healthy individuals (such as organ donors), thereby improving their applicability across diverse populations.

For sarcopenia, the age- and sex-specific cut-off values corresponding to the lower 25th percentile of skeletal muscle index (SMI) from the population-representative study by Weston et al. were applied to our cohort (**Supplemental Table 1**). Patients falling below their age- and sex-specific threshold were classified as sarcopenic.

The presence of myosteatosis (decreased muscle quality due to intramuscular infiltration of ectopic adipose tissue) was determined based on the skeletal muscle density (SMD), following the same methodology used for sarcopenia. Accordingly, patients with skeletal muscle density below the age-

and sex-specific 25th percentile in the population-representative study by Weston et al. were classified as having myosteatosis (**Supplemental Table 1**).

Statistical Analysis

Continuous data are reported as medians with interquartile ranges (IQR), while categorical data are presented as absolute frequencies and percentages.

The assumptions of the Cox proportional hazards model were assessed using the Schoenfeld residuals test. Linearity of continuous variables was assessed. Multivariable analyses with obesity or sarcopenic obesity as the primary independent variable were not additionally adjusted for BMI.

For continuous variables measured in cm^2, cm^2/m^2 or Hounsfield units, hazard ratios (HRs) are reported per 30-unit decrease. This approach was chosen to enhance interpretability, as, for example, subcutaneous adipose tissue has a median of 182.1 cm^2 (IQR 127.8–258.0). Reporting HRs per 1 cm^2 change would yield extremely small values with multiple decimal places, reducing clarity and readability. Furthermore, we opted for a 30-unit decrease rather than an increase, as presenting HRs per 30-unit increase would result in HRs below 1, which may be counterintuitive in the context of higher mortality and worse survival.

Statistical analyses were conducted using the survival package and forestplot package in R (version 4.3.2) within RStudio (version 2024.04.0).

SUPPLEMENTAL TABLE 1

Age- and Sex-Specific Cut-Off Values for the Presence of Sarcopenia and Myosteatosis

Body Composition Parameter	Men	Women
**Sarcopenia**		
20-24 years old	SMI < 52.1 cm^2/m^2	SMI < 45.5 cm^2/m^2
25-29 years old	SMI < 54.0 cm^2/m^2	SMI < 46.1 cm^2/m^2
30-34 years old	SMI < 55.7 cm^2/m^2	SMI < 46.7 cm^2/m^2
35-39 years old	SMI < 56.8 cm^2/m^2	SMI < 46.9 cm^2/m^2
40-44 years old	SMI < 57.4 cm^2/m^2	SMI < 46.8 cm^2/m^2
45-49 years old	SMI < 57.5 cm^2/m^2	SMI < 46.5 cm^2/m^2
50-54 years old	SMI < 57.0 cm^2/m^2	SMI < 45.9 cm^2/m^2
55-59 years old	SMI < 56.1 cm^2/m^2	SMI < 45.1 cm^2/m^2
60-64 years old	SMI < 55.0 cm^2/m^2	SMI < 44.3 cm^2/m^2
65-69 years old	SMI < 53.7 cm^2/m^2	SMI < 43.4 cm^2/m^2
70-74 years old	SMI < 52.2 cm^2/m^2	SMI < 42.4 cm^2/m^2
75-79 years old	SMI < 50.5 cm^2/m^2	SMI < 41.8 cm^2/m^2
80-84 years old	SMI < 48.6 cm^2/m^2	SMI < 41.8 cm^2/m^2
>85 years old	SMI < 46.5 cm^2/m^2	SMI < 42.4 cm^2/m^2
**Myosteatosis**		
20-24 years old	SMD < 40.3 HU	SMD < 32.4 HU
25-29 years old	SMD < 36.8 HU	SMD < 29.2 HU
30-34 years old	SMD < 34.1 HU	SMD < 27.2 HU
35-39 years old	SMD < 31.7 HU	SMD < 25.6 HU
40-44 years old	SMD < 29.2 HU	SMD < 23.7 HU
45-49 years old	SMD < 26.8 HU	SMD < 21.4 HU
50-54 years old	SMD < 24.4 HU	SMD < 18.9 HU
55-59 years old	SMD < 21.9 HU	SMD < 16.1 HU
60-64 years old	SMD < 19.3 HU	SMD < 13.3 HU
65-69 years old	SMD < 16.6 HU	SMD < 10.6 HU
70-74 years old	SMD < 13.8 HU	SMD < 7.8 HU
75-79 years old	SMD < 10.6 HU	SMD < 5.1 HU
80-84 years old	SMD < 7.2 HU	SMD < 2.4 HU
>85 years old	SMD < 3.3 HU	SMD < –0.3 HU

Caption

Age- and sex-specific cut-off values used to determine the presence of sarcopenia and myosteatosis in patients with pancreatic ductal adenocarcinoma, based on Weston et al. (2024).

Abbreviations: HU, Hounsfield units; SMD, skeletal muscle density; SMI, skeletal muscle index.

SUPPLEMENTAL TABLE 2

Age- and Sex-Specific Distribution of Body Composition Parameters

	Patients	BMI (kg/m²)	Obesity	SMI (cm²/m²)	Sarcopenia	Sarcopenic obesity	SMD (HU)	Myo-steatosis	VAT (cm²)	SAT (cm²)	BA (cm²)	BD (HU)
**Male**												
30-34 years old	3	21.1<br>(18.5 – 23.1)	0<br>(0.0%)	48.4<br>(42.0 – 51.5)	3<br>(100.0%)	0<br>(0.0%)	53.2<br>(47.3 – 54.6)	0<br>(0.0%)	62.3<br>(32.3 – 66.3)	78.1<br>(45.2 – 100.3)	29.6<br>(24.9 – 31.2)	312.7<br>(308.2 – 344.7)
35-39 years old	6	26.6<br>(23.1 – 28.1)	0<br>(0.0%)	55.1<br>(48.3 – 59.1)	3<br>(50.0%)	0<br>(0.0%)	39.3<br>(33.9 – 43.0)	0<br>(0.0%)	75.3<br>(46.9 – 97.8)	147.6<br>(89.0 – 219.3)	28.2<br>(26.0 – 38.8)	328.4<br>(318.6 – 367.2)
40-44 years old	14	24.6<br>(21.4 – 26.3)	0<br>(0.0%)	48.7<br>(41.4 – 57.5)	10<br>(71.4%)	0<br>(0.0%)	32.0<br>(27.7 – 38.7)	4<br>(28.6%)	90.4<br>(61.4 – 134.5)	156.8<br>(78.3 – 217.7)	28.5<br>(24.9 – 33.1)	330.6<br>(302.5 – 359.3)
45-49 years old	33	26.5<br>(22.4 – 31.5)	12<br>(36.4%)	55.9<br>(49.5 – 63.7)	19<br>(57.6%)	4<br>(12.1%)	32.7<br>(24.1 – 41.4)	10<br>(30.3%)	150.8<br>(66.2 – 193.2)	154.8<br>(116.0 – 277.0)	34.9<br>(28.5 – 45.6)	289.2<br>(274.5 – 326.5)
50-54 years old	73	27.1<br>(24.4 – 29.4)	16<br>(21.9%)	54.6<br>(49.9 – 61.1)	45<br>(61.6%)	2<br>(2.7%)	31.1<br>(25.9 – 38.1)	13<br>(17.8%)	179.9<br>(93.6 – 231.2)	171.7<br>(129.2 – 227.0)	31.6<br>(27.7 – 39.5)	307.1<br>(274.9 – 332.8)
55-59 years old	101	26.8<br>(24.8 – 31.9)	30<br>(29.7%)	54.6<br>(49.9 – 60.1)	57<br>(56.4%)	6<br>(5.9%)	29.6<br>(21.9 – 35.0)	26<br>(25.7%)	177.6<br>(99.6 – 247.1)	182.6<br>(131.4 – 262.4)	33.7<br>(28.9 – 41.7)	290.9<br>(271.7 – 328.7)
60-64 years old	124	27.7<br>(24.6 – 30.3)	35<br>(28.2%)	53.1<br>(48.0 – 59.8)	72<br>(58.1%)	12<br>(9.7%)	29.1<br>(24.2 – 33.8)	21<br>(16.9%)	180.6<br>(97.1 – 232.9)	168.0<br>(115.0 – 236.6)	34.7<br>(29.4 – 43.1)	294.4<br>(266.4 – 327.2)
65-69 years old	144	27.2<br>(24.0 – 29.7)	33<br>(22.9%)	52.1<br>(47.4 – 57.5)	79<br>(54.9%)	8<br>(5.6%)	24.3<br>(17.8 – 32.2)	32<br>(22.2%)	181.0<br>(113.7 – 253.5)	173.7<br>(119.5 – 232.4)	36.4<br>(29.8 – 43.6)	288.5<br>(265.4 – 324.9)
70-74 years old	161	26.4<br>(24.1 – 28.9)	33<br>(20.5%)	51.0<br>(45.3 – 56.3)	87<br>(54.0%)	7<br>(4.3%)	23.6<br>(16.4 – 30.3)	27<br>(16.8%)	172.5<br>(103.0 – 234.7)	165.1<br>(119.6 – 213.7)	37.9<br>(31.3 – 47.2)	290.0<br>(254.4 – 324.4)
75-79 years old	145	26.6<br>(24.2 – 30.0)	37<br>(25.5%)	52.0<br>(48.0 – 57.0)	61<br>(42.1%)	7<br>(4.8%)	19.7<br>(13.3 – 26.8)	29<br>(20.0%)	197.2<br>(110.8 – 270.7)	167.7<br>(127.5 – 238.0)	40.3<br>(32.1 – 49.8)	279.6<br>(250.7 – 317.3)
80-84 years old	93	26.0<br>(23.3 – 28.0)	12<br>(12.9%)	51.9<br>(47.0 – 55.6)	32<br>(34.4%)	2<br>(2.2%)	19.4<br>(12.2 – 25.0)	13<br>(14.0%)	175.4<br>(107.6 – 241.5)	164.6<br>(122.6 – 212.5)	42.1<br>(33.8 – 48.1)	264.1<br>(243.5 – 290.4)
>85 years old	41	25.2<br>(23.7 – 28.7)	5<br>(12.2%)	49.2<br>(45.3 – 53.4)	14<br>(34.1%)	0<br>(0.0%)	18.0<br>(10.9 – 23.2)	3<br>(7.3%)	189.1<br>(113.5 – 244.6)	155.4<br>(121.2 – 183.6)	42.9<br>(35.2 – 46.1)	254.0<br>(226.6 – 282.3)
**Female**												
30-34 years old	2	32.0<br>(28.2 – 35.9)	1<br>(50.0%)	48.9<br>(46.6 – 51.3)	1<br>(50.0%)	0<br>(0.0%)	29.1<br>(25.8 – 32.4)	1<br>(50.0%)	91.6<br>(73.3 – 109.9)	327.3<br>(243.4 – 411.2)	33.0<br>(32.8 – 33.3)	324.1<br>(317.5 – 330.6)
35-39 years old	7	25.7<br>(23.5 – 26.0)	1<br>(14.3%)	56.2<br>(45.8 – 59.0)	2<br>(28.6%)	0<br>(0.0%)	35.4<br>(31.3 – 37.4)	1<br>(14.3%)	20.8<br>(10.3 – 63.8)	176.0<br>(150.1 – 268.9)	28.8<br>(22.5 – 37.2)	331.8<br>(321.8 – 382.2)
40-44 years old	17	25.8<br>(22.3 – 29.5)	4<br>(23.5%)	46.9<br>(43.5 – 53.8)	8<br>(47.1%)	0<br>(0.0%)	33.6<br>(30.2 – 38.1)	4<br>(23.5%)	68.4<br>(24.9 – 112.7)	187.3<br>(135.4 – 290.6)	27.6<br>(23.4 – 34.8)	317.9<br>(305.7 – 339.2)
45-49 years old	12	24.5<br>(21.9 – 31.0)	4<br>(33.3%)	44.7<br>(41.3 – 49.2)	8<br>(66.7%)	1<br>(8.3%)	31.0<br>(29.0 – 41.5)	1<br>(8.3%)	49.5<br>(10.7 – 120.5)	205.0<br>(90.6 – 318.3)	25.5<br>(21.6 – 34.2)	306.9<br>(285.2 – 336.2)
50-54 years old	38	25.6<br>(21.6 – 28.7)	7<br>(18.4%)	45.3<br>(42.6 – 48.9)	22<br>(57.9%)	1<br>(2.6%)	26.1<br>(18.1 – 30.5)	10<br>(26.3%)	107.4<br>(58.3 – 131.6)	207.2<br>(157.8 – 314.0)	25.9<br>(21.9 – 30.9)	315.6<br>(270.7 – 360.0)
55-59 years old	68	27.4<br>(23.0 – 31.6)	21<br>(30.9%)	45.8<br>(41.1 – 51.3)	33<br>(48.5%)	3<br>(4.4%)	22.6<br>(16.7 – 26.9)	16<br>(23.5%)	90.2<br>(55.5 – 135.8)	245.4<br>(172.4 – 330.0)	31.2<br>(25.2 – 37.1)	283.1<br>(261.9 – 318.1)
60-64 years old	76	24.1<br>(21.3 – 29.4)	18<br>(23.7%)	44.6<br>(39.2 – 49.9)	37<br>(48.7%)	2<br>(2.6%)	22.6<br>(15.9 – 30.0)	16<br>(21.1%)	93.7<br>(52.8 – 133.7)	208.2<br>(150.9 – 280.6)	28.5<br>(22.6 – 36.2)	292.4<br>(267.7 – 327.7)
65-69 years old	122	25.5<br>(21.7 – 32.3)	38<br>(31.1%)	45.8<br>(41.4 – 51.3)	42<br>(34.4%)	3<br>(2.5%)	21.2<br>(12.1 – 28.8)	27<br>(22.1%)	91.6<br>(38.5 – 154.4)	235.6<br>(143.3 – 351.1)	30.5<br>(24.7 – 37.6)	274.3<br>(252.6 – 299.2)
70-74 years old	131	26.2<br>(22.3 – 28.8)	26<br>(19.8%)	44.6<br>(39.3 – 49.0)	52<br>(39.7%)	1<br>(0.8%)	17.9<br>(9.6 – 25.0)	26<br>(19.8%)	93.3<br>(47.0 – 141.5)	209.6<br>(155.1 – 278.6)	31.0<br>(25.6 – 37.7)	282.1<br>(247.1 – 308.3)
75-79 years old	131	25.9<br>(22.6 – 29.0)	31<br>(23.7%)	45.8<br>(39.1 – 50.7)	43<br>(32.8%)	5<br>(3.8%)	13.5<br>(8.4 – 24.1)	23<br>(17.6%)	78.6<br>(45.4 – 142.2)	203.0<br>(138.7 – 292.9)	30.0<br>(25.0 – 37.9)	260.7<br>(235.7 – 290.2)
80-84 years old	77	25.5<br>(22.9 – 28.3)	13<br>(16.9%)	43.7<br>(39.7 – 47.5)	30<br>(39.0%)	1<br>(1.3%)	13.3<br>(4.0 – 19.6)	17<br>(22.1%)	92.1<br>(44.7 – 123.8)	173.6<br>(132.5 – 240.7)	31.3<br>(26.5 – 39.8)	267.5<br>(231.9 – 301.0)
>85 years old	47	25.4<br>(22.6 – 29.1)	10<br>(21.3%)	45.8<br>(42.4 – 50.6)	12<br>(25.5%)	1<br>(2.1%)	13.4<br>(6.3 – 18.4)	8<br>(17.0%)	94.8<br>(45.6 – 139.6)	182.6<br>(115.6 – 244.5)	34.4<br>(30.0 – 39.8)	241.6<br>(216.2 – 264.5)

Caption

Distribution of body composition parameters in the overall cohort, stratified by sex and age group. Continuous variables are reported as median with interquartile range (IQR), and categorical variables are reported as absolute frequencies with percentages.

Abbreviations: BA, bone area; BD, bone density; BMI, body mass index; HU, Hounsfield units; IQR, interquartile range; SAT, subcutaneous adipose tissue area; SMD, skeletal muscle density; SMI, skeletal muscle index; VAT, visceral adipose tissue area.

SUPPLEMENTAL TABLE 3

Table 3. Breakdown of the Charlson Comorbidity Index (CCI) for All PDAC Patients and Treatment Subgroups

	All patients (n=1666)	Surgery (n=509)	Palliative therapy (n=439)	No treatment (n=718)
**Age**	69 (61 – 76)	66 (58 – 73)	68 (60 – 75)	72.5 (64 – 79)
50-59 years	280 (16.8%)	117 (23.0%)	80 (18.2%)	83 (11.6%)
60-69 years	466 (28.0%)	150 (29.5%)	134 (30.5%)	182 (25.3%)
70-79 years	568 (34.1%)	163 (32.0%)	151 (34.4%)	254 (35.4%)
≥80 years	258 (15.5%)	39 (7.7%)	49 (11.2%)	170 (23.7%)
**Myocardial infarction**	571 (34.3%)	248 (48.7%)	171 (39.0%)	152 (21.2%)
**Congestive heart failure**	129 (7.7%)	52 (10.2%)	41 (9.3%)	36 (5.0%)
**Peripheral vascular disease**	314 (18.8%)	161 (31.6%)	100 (22.8%)	53 (7.4%)
**CVA or TIA**	96 (5.8%)	42 (8.3%)	29 (6.6%)	25 (3.5%)
**Dementia**	22 (1.3%)	8 (1.6%)	4 (0.9%)	10 (1.4%)
**COPD**	250 (15.0%)	113 (22.2%)	72 (16.4%)	65 (9.1%)
**Connective tissue disease**	47 (2.8%)	18 (3.5%)	12 (2.7%)	17 (2.4%)
**Peptic ulcer disease**	110 (6.6%)	52 (10.2%)	35 (8.0%)	23 (3.2%)
**Liver disease**				
Mild	537 (32.2%)	237 (46.6%)	155 (35.3%)	145 (20.2%)
Moderate to severe	96 (5.8%)	42 (8.3%)	31 (7.1%)	23 (3.2%)
**Diabetes mellitus**				
Uncomplicated	411 (24.7%)	163 (32.0%)	95 (21.6%)	153 (21.3%)
End-organ damage	164 (9.8%)	84 (16.5%)	48 (10.9%)	32 (4.5%)
**Hemiplegia**	13 (0.8%)	5 (1.0%)	3 (0.7%)	5 (0.7%)
**Moderate to severe CKD**	146 (8.8%)	60 (11.8%)	37 (8.4%)	49 (6.8%)
**Solid tumor**				
Localized	1084 (65.1%	484 (95.1%)	220 (50.1%)	380 (52.9%)
Metastatic	582 (34.9%)	25 (4.9%)	219 (49.9%)	338 (47.1%)
**Leukemia**	0 (0.0%)	0 (0.0%)	0 (0.0%)	0 (0.0%)
**Lymphoma**	28 (1.7%)	12 (2.4%)	6 (1.4%)	10 (1.4%)
**AIDS**	0 (0.0%)	0 (0.0%)	0 (0.0%)	0 (0.0%)
**CCI**	8 (5 – 9)	7 (5 – 8)	8 (6 – 10)	8 (6 – 10)
**Modified CCI**	1 (0 – 2)	1 (1 – 3)	1 (0 – 2)	0 (0 – 1)

Caption

Pre-existent comorbidities were assessed using International Classification of Diseases (ICD-9 and ICD-10) codes and aggregated in a modified version of the Charlson Comorbidity Index (CCI) excluding age, diabetes and tumor stage. Continuous variables are reported as median with interquartile range (IQR), and categorical variables are reported as absolute frequencies with percentages.

Abbreviations: AIDS, acquired immunodeficiency syndrome; CKD, chronic kidney disease; CVA, cerebrovascular accident; COPD, chronic obstructive pulmonary disease; TIA, transient ischemic attack.

SUPPLEMENTAL TABLE 4

Table 4. Detailed Treatment Characteristics of Patients with PDAC

	**Surgery (n=509)**	**Palliative therapy (n=439)**
**Type of surgery**		
Pancreatoduodenectomy	364 (71.5%)	
Partial pancreatectomy	109 (21.4%)	
Total pancreatectomy	10 (2.0%)	
Unknown	26 (5.1%)	
**Surgical approach**		
Open approach	176 (34.6%)	
Endoscopic approach	93 (18.3%)	
Endoscopic converted to open	13 (2.6%)	
Robotic-assisted	3 (0.6%)	
Unknown	224 (44.0%)	
**Surgical margin status**		
No residual tumor (R0)	410 (80.6%)	
Microscopic residual tumor (R1)	39 (7.7%)	
Macroscopic residual tumor (R2)	2 (0.4%)	
Residual tumor, unspecified (R1/R2)	12 (2.4%)	
Unknown	46 (9.0%)	
**Sequence of surgery and radiotherapy**		
Radiotherapy before surgery	75 (14.7%)	
Radiotherapy after surgery	113 (22.2%)	
Radiotherapy, sequence unknown	8 (1.6%)	
Not applicable (no radiotherapy)	313 (61.5%)	
**Radiotherapy administration**		
Beam radiation	181 (35.6%)	112 (25.5%)
Radiotherapy, unspecified	15 (2.9%)	4 (0.9%)
Not applicable (no radiotherapy)	313 (61.5%)	323 (73.6%)
**Sequence of surgery and systemic therapy**		
Systemic therapy before surgery (neoadjuvant)	78 (15.3%)	
Systemic therapy after surgery (adjuvant)	182 (35.8%)	
Systemic therapy before and after surgery	30 (5.9%)	
Unknown	47 (9.2%)	
Not applicable (no systemic therapy)	172 (33.8%)	
**Chemotherapy administration**		
Single-agent chemotherapy	173 (34.0%)	157 (35.8%)
Multi-agent chemotherapy	158 (31.0%)	258 (58.8%)
Chemotherapy, unspecified	6 (1.2%)	7 (1.6%)
Unknown	42 (8.3%)	0 (0.0%)
Not applicable (no chemotherapy)	130 (25.5%)	17 (3.9%)

Caption

Summary of surgical procedures, surgical margin status, chemotherapy and radiotherapy administration, and treatment sequencing in the study cohort.

SUPPLEMENTAL TABLE 5

Table 5. Imaging Acquisition and Reconstruction Parameters

**Slice Thickness**	**Count (percentage)**
<1 mm	8 (0.5%)
2-3 mm	638 (38.1%)
3-4 mm	194 (11.6%)
4-5 mm	3 (0.2%)
5-6 mm	510 (30.4%)
6-7 mm	6 (0.4%)
7-8 mm	16 (1.0%)
8-9 mm	2 (0.1%)
>=10 mm	27 (1.6%)
Other / Not specified	271 (16.2%)
**Manufacturer**	
Siemens	876 (52.3%)
GE	420 (25.1%)
Toshiba	84 (5.0%)
Philips	19 (1.1%)
Other / Not specified	276 (16.5%)
**Tube Voltage (kVp)**	
80	13 (0.8%)
90	11 (0.7%)
100	206 (12.3%)
110	2 (0.1%)
120	1112 (66.4%)
130	20 (1.2%)
135	2 (0.1%)
140	40 (2.4%)
Other / Not specified	269 (16.1%)
**Convolution Kernel**	
B40f	580 (34.6%)
Standard	403 (24.1%)
I30f	180 (10.7%)
FC13	41 (2.4%)
Br44d	34 (2.0%)
B30f	26 (1.6%)
FC18	21 (1.3%)
B31f	20 (1.2%)
I40f	8 (0.5%)
'Normal'	6 (0.4%)
Other / Not specified	356 (21.3%)
**Year**	
2000	18 (1.1%)
2001	15 (0.9%)
2002	44 (2.6%)
2003	61 (3.6%)
2004	73 (4.4%)
2005	74 (4.4%)
2006	61 (3.6%)
2007	95 (5.7%)

2008	109 (6.5%)
2009	86 (5.1%)
2010	87 (5.2%)
2011	99 (5.9%)
2012	81 (4.8%)
2013	78 (4.7%)
2014	84 (5.0%)
2015	70 (4.2%)
2016	76 (4.5%)
2017	84 (5.0%)
2018	91 (5.4%)
2019	12 (0.7%)
2020	8 (0.5%)
Other / Not specified	269 (16.1%)

Caption

Summary of key CT imaging parameters for all scans included in the study, including slice thickness, scanner manufacturer, tube voltage (kVp), convolution kernel, and year of acquisition. Due to anonymization of the CT scans, detailed acquisition parameters could not be retrieved for 269 patients (“Not specified”). In addition, nine instances of duplicate Series Descriptions resulted in a total of 1675 series instead of 1666.

<u>**SUPPLEMENTAL FIGURE 1**</u>

Title

Supplemental Figure 1. Kaplan-Meier Curve for Overall Survival of Patients with PDAC Stratified by Treatment Subgroups

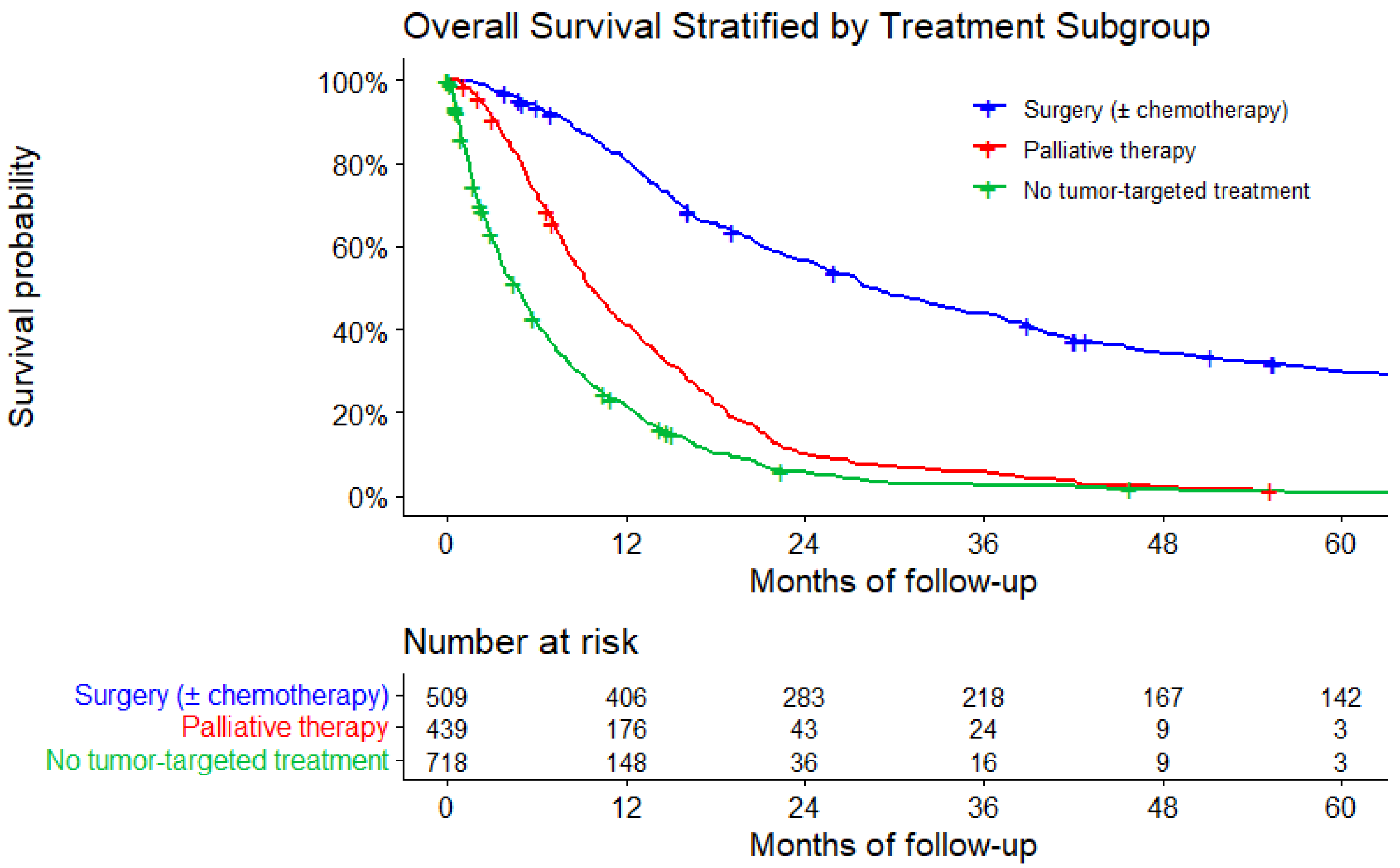

Caption

Kaplan-Meier Curve for Overall Survival Stratified by Treatment Subgroups of patients who underwent surgery (mOS 28.3 months, 95% CI 25.5 – 33.4), received palliative therapy (mOS 9.7 months, 95% CI 8.9 – 10.8), or did not undergo tumor-targeted treatment (mOS 4.8 months, 95% CI 3.9 – 5.3).

Abbreviations: CI, confidence interval; mOS, median overall survival; PDAC, pancreatic ductal adenocarcinoma.

SUPPLEMENTAL FIGURE 2

Supplemental Figure 2. Kaplan-Meier Curves of Patients with PDAC Who Underwent Surgery

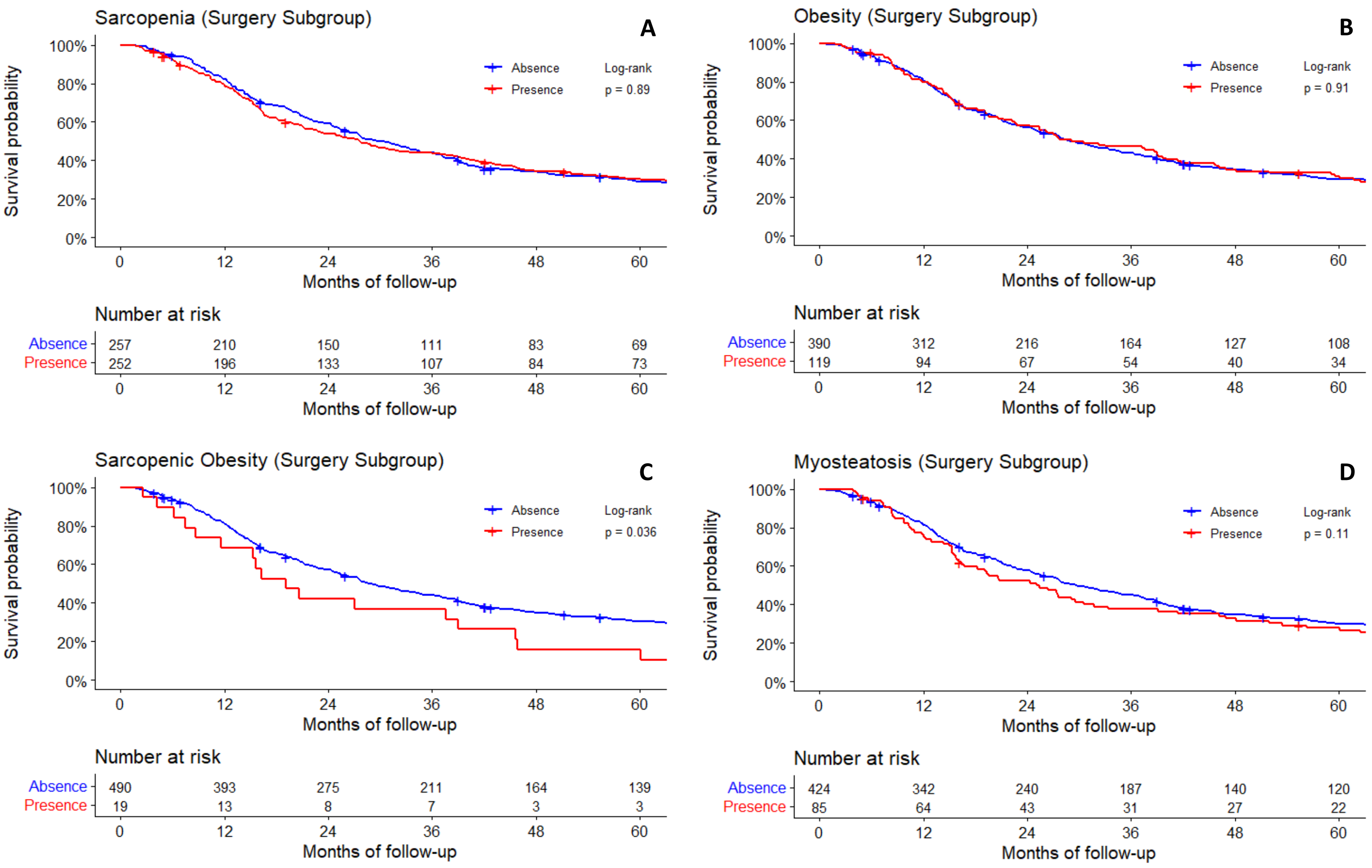

Caption

Kaplan-Meier survival curves of patients with PDAC who underwent surgery, stratified by the presence of sarcopenia (a), obesity (b), sarcopenic obesity (c), and myosteatosis (d). The analysis showed significantly worse survival for patients with sarcopenic obesity (mOS 19.1 [95% CI 15.3 – 45.8] versus 28.9 [95% CI 26.2 – 34.1] months, P=0.04). There were no significant survival differences for patients with sarcopenia (mOS 27.6 [95% CI 22.3 – 36.9] versus 29.7 [95% CI 25.5 – 36.3] months, P=0.89), obesity (mOS 27.7 [95% CI 22.9 – 40.1] versus 28.3 [95% CI 24.9 – 33.5] months, P=0.91), and myosteatosis (mOS 25.1 [95% CI 16.7 – 33.4] versus 29.7 [95% CI 26.2 – 36.3] months, P=0.11).

Abbreviations: CI, confidence interval; mOS, median overall survival; PDAC, pancreatic ductal adenocarcinoma.

SUPPLEMENTAL FIGURE 3

Supplemental Figure 3. Kaplan-Meier Curves of Patients with PDAC Who Received Palliative Therapy

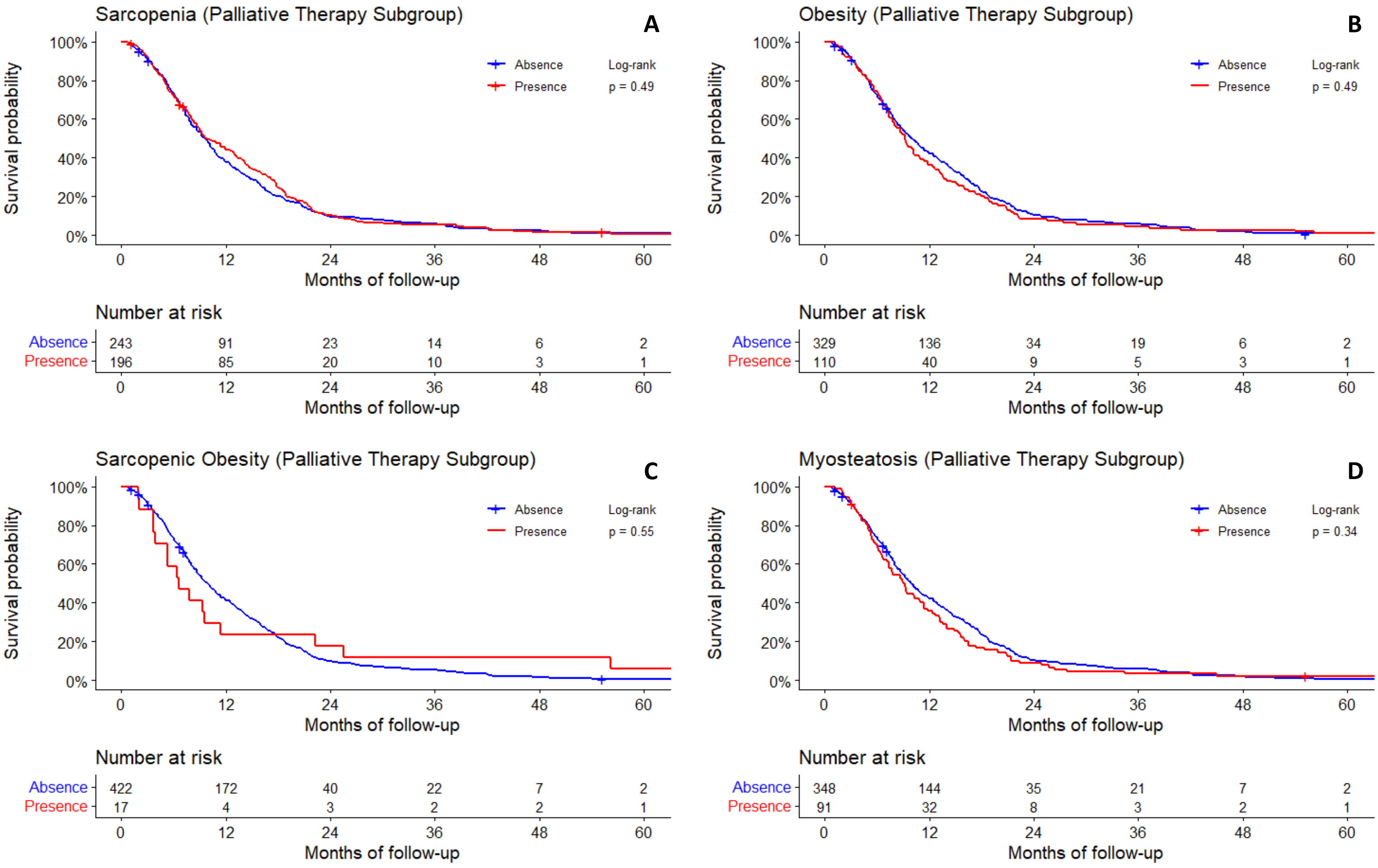

Caption

Kaplan-Meier survival curves of patients with PDAC who received palliative therapy, stratified by the presence of sarcopenia (a), obesity (b), sarcopenic obesity (c), and myosteatosis (d). The analysis showed no significant survival differences for patients with sarcopenia (mOS 9.7 [95% CI 8.7 – 12.9] versus 9.6 [95% CI 8.4 – 10.7] months, P=0.49), obesity (mOS 9.2 [95% CI 7.8 – 11.0] versus 10.0 [95% CI 8.8 – 11.4] months, P=0.49), sarcopenic obesity (mOS 6.6 [95% CI 5.3 – 22.3] versus 9.9 [95% CI 9.0 – 10.9] months, P=0.55), and myosteatosis (mOS 9.2 [95% CI 7.4 – 11.4] versus 10.0 [95% CI 8.9 – 11.2] months, P=0.34).

Abbreviations: CI, confidence interval; mOS, median overall survival; PDAC, pancreatic ductal adenocarcinoma.

SUPPLEMENTAL FIGURE 4

Supplemental Figure 4. Kaplan-Meier Curves of Patients with PDAC Who Did Not Undergo Tumor-Targeted Treatment

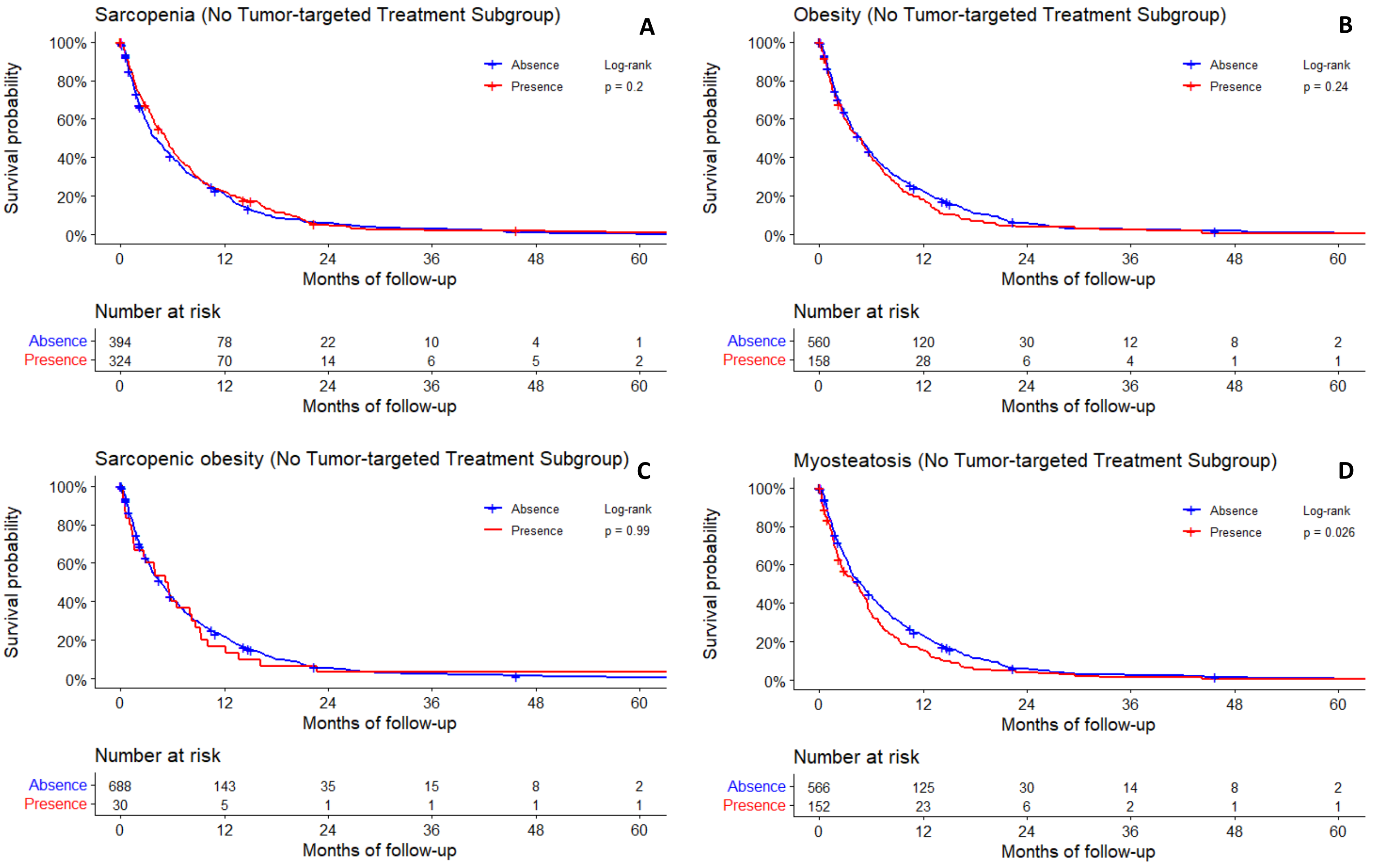

Caption

Kaplan-Meier survival curves of patients with PDAC who did not undergo tumor-targeted treatment, stratified by the presence of sarcopenia (a), obesity (b), sarcopenic obesity (c), and myosteatosis (d). The analysis showed significantly worse survival for patients with myosteatosis (mOS 4.2 [95% CI 2.8 – 5.6] versus 4.8 [95% CI 3.9 – 5.7] months, P=0.03). There were no significant survival differences for patients with sarcopenia (mOS 5.3 [95% CI 4.4 – 6.0] versus 3.9 [95% CI 3.4 – 5.0] months, P=0.20), obesity (mOS 4.7 [95% CI 3.4 – 5.8] versus 4.8 [95% CI 3.9 – 5.6] months,

P=0.24), and sarcopenic obesity (mOS 5.3 [95% CI 2.7 – 8.6] versus 4.7 [95% CI 3.9 – 5.3] months, P=0.99).

Abbreviations: CI, confidence interval; mOS, median overall survival; PDAC, pancreatic ductal adenocarcinoma.

SUPPLEMENTAL FIGURE 5

Supplemental Figure 5. Hazard Ratios for Mortality in PDAC Patients Stratified by Subgroup

Body composition parameter	Subgroup	HR (95% CI)	P-value
Body mass index	surgery	1.00 (0.98 – 1.02)	0.67
HR for every 1 kg/m² increase	palliative	1.01 (0.99 – 1.03)	0.20
	no treatment	1.01 (0.99 – 1.02)	0.50
Obesity	surgery	1.06 (0.83 – 1.34)	0.65
HR for presence of	palliative	1.15 (0.91 – 1.47)	0.24
	no treatment	1.14 (0.94 – 1.39)	0.19
Skeletal muscle index	surgery	1.04 (0.68 – 1.59)	0.87
HR for every 30 cm²/m² decrease	palliative	1.00 (0.69 – 1.46)	1.00
	no treatment	0.92 (0.67 – 1.28)	0.63
Sarcopenia	surgery	1.04 (0.82 – 1.32)	0.76
HR for presence of	palliative	1.02 (0.81 – 1.28)	0.86
	no treatment	1.03 (0.87 – 1.22)	0.71
Sarcopenic obesity	surgery	1.75 (1.06 – 2.91)	0.03
HR for presence of	palliative	0.92 (0.54 – 1.57)	0.76
	no treatment	1.13 (0.76 – 1.68)	0.55
Skeletal muscle density	surgery	1.26 (0.87 – 1.81)	0.22
HR for every 30 Hounsfield units decrease	palliative	1.43 (1.03 – 1.99)	0.03
	no treatment	1.12 (0.86 – 1.45)	0.39
Myosteatosis	surgery	1.56 (1.16 – 2.11)	0.004
HR for presence of	palliative	1.18 (0.91 – 1.53)	0.22
	no treatment	1.09 (0.88 – 1.36)	0.42
Visceral adipose tissue area	surgery	0.99 (0.95 – 1.04)	0.74
HR for every 30 cm² decrease	palliative	1.00 (0.96 – 1.05)	0.92
	no treatment	1.04 (1.01 – 1.08)	0.02
Subcutaneous adipose tissue area	surgery	1.09 (1.03 – 1.16)	0.002
HR for every 30 cm² decrease	palliative	1.01 (0.96 – 1.06)	0.69
	no treatment	1.01 (0.96 – 1.05)	0.80
Bone area	surgery	0.72 (0.49 – 1.05)	0.08
HR for every 30 cm² decrease	palliative	0.86 (0.60 – 1.23)	0.42
	no treatment	0.67 (0.52 – 0.86)	0.001
Bone density	surgery	1.05 (0.99 – 1.12)	0.13
HR for every 30 Hounsfield units decrease	palliative	1.01 (0.95 – 1.07)	0.79
	no treatment	1.05 (1.00 – 1.10)	0.07

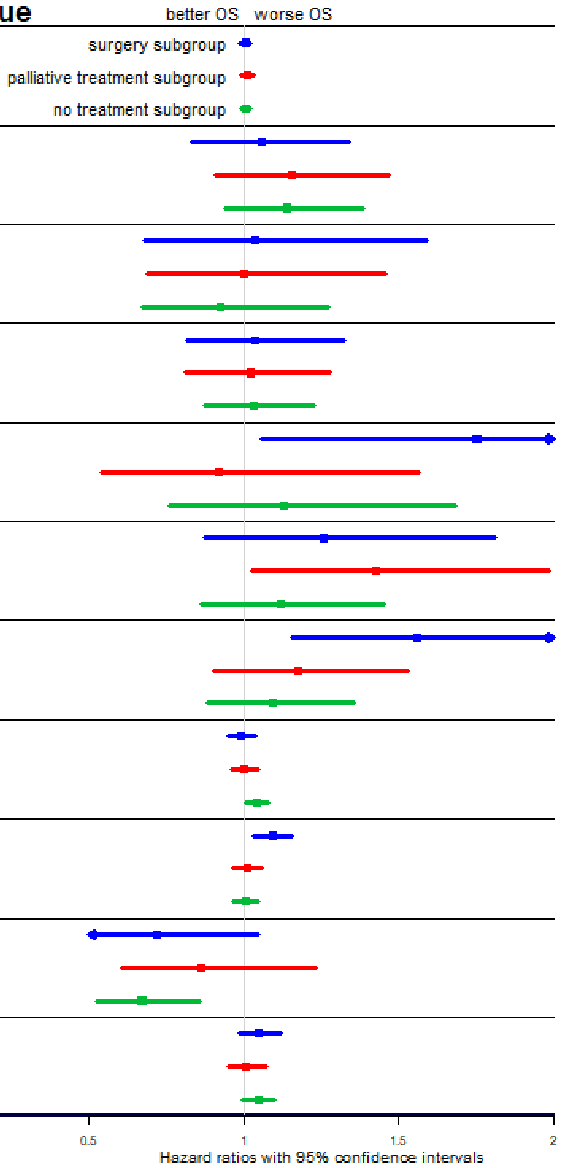

Caption

Forest plot showing hazard ratios for mortality and their 95% confidence intervals in patients with PDAC, stratified by treatment group: surgical resection (blue lines), palliative therapy (red lines), and no tumor-targeted treatment (green lines). All results are determined using multivariable Cox regression analysis. To study the independent association between various body composition

parameters and overall survival, separate multivariable analyses were performed with every body composition parameter individually adjusted for potential confounders (age, sex, race/ethnicity, alcohol consumption, smoking status, ECOG Performance Status, comorbidities, diabetes, tumor localization, tumor stage, and CA19-9 level).

Abbreviations: BMI, body mass index; CA19-9, cancer antigen 19-9; CCI, Charlson Comorbidity Index; CI, confidence interval; ECOG, Eastern Cooperative Oncology Group; HR, hazard ratio; OS, overall survival; PDAC, pancreatic ductal adenocarcinoma.
